# Prevalence and Diagnosis of Obstructive Sleep Apnea in Atrial Fibrillation Patients: A Systematic Review

**DOI:** 10.3390/jcm14165708

**Published:** 2025-08-12

**Authors:** Susana Sousa, Marta Drummond, António Bugalho

**Affiliations:** 1 Sleep Unit, CUF Tejo Hospital and CUF Descobertas Hospital, 1998-018 Lisbon, Portugal; 2 Pulmonology Department, CUF Tejo Hospital, 1998-018 Lisbon, Portugal; antonio.bugalho@cuf.pt; 3 NOVA Medical School, Nova University of Lisbon, 1169-056 Lisbon, Portugal; 4 Comprehensive Health Research Center (CHRC), 1150-082 Lisbon, Portugal; 5 Sleep and Non-Invasive Unit, Centro Hospitalar e Universitário São João, 4200-319 Porto, Portugal; marta.drummond@gmail.com; 6 Faculty of Medicine, University of Porto, 4200-319 Porto, Portugal

**Keywords:** atrial fibrillation, cardiac arrhythmias, cardiovascular comorbidity, cardiovascular disease, diagnosis, home sleep apnea testing, obstructive sleep apnea, prevalence, sleep-disordered breathing, polysomnography, risk factors, systematic review

## Abstract

Obstructive sleep apnea (OSA) is a highly prevalent and underdiagnosed sleep disorder with significant cardiovascular implications, namely in atrial fibrillation (AF) patients. Despite its clinical relevance, OSA prevalence among AF patients and the diagnostic strategies used remain heterogeneous across studies, complicating screening, and treatment pathways. Our aim was to synthesize recent evidence on OSA prevalence in AF populations and to critically evaluate the diagnostic methods and screening strategies employed in clinical studies, by conducting a systematic review using PubMed and Google Scholar to identify original clinical studies published between January-2019 and December-2024. Inclusion criteria targeted adult AF populations assessed for OSA or sleep-disordered breathing. The results were analyzed by two independent reviewers. Non-concordances were resolved by consensus. Data extracted included study characteristics, population profiles, diagnostic approaches, prevalence rates, symptom profiles, and clinical correlates. Thirty-eight studies were included, comprising predominantly observational studies. Prevalence estimates of OSA in AF populations ranged from 5% to 90%, with most studies reporting rates > 60%. A consistent burden of moderate-to-severe OSA was observed. Diagnostic methods varied widely, from polysomnography (PSG) and home sleep apnea testing to pacemaker-derived monitoring and questionnaires such as STOP-Bang and Epworth Sleepiness Scale (ESS). Underdiagnosis was attributed to minimal symptomatology, lack of physician awareness, and reliance on subjective tools. Several studies highlighted the limited sensitivity of standard screening instruments in AF populations and advocated for objective testing even in asymptomatic patients. Marked heterogeneity in study designs, diagnostic methods, and populations precluded quantitative synthesis and limited direct comparisons. Objective diagnostic testing, particularly PSG, is essential to improve OSA detection rates and guide individualized management. Integration of structured screening protocols into AF care—especially for high-risk patients—and interdisciplinary collaboration are critical.

## 1. Introduction

The link between cardiovascular diseases and sleep-related breathing disorders (SDBs) has been recognized for a long time. Among these, obstructive sleep apnea (OSA) is the most prevalent and has received increasing attention from the medical community due to its clinical significance [1]. OSA is characterized by repeated episodes of upper airway obstruction during sleep, resulting in intermittent apnea and hypopnea, and frequent episodes of decreased arterial oxygen saturation. It is typically diagnosed when the apnea–hypopnea index (AHI) is ≥5 (AHI is used to classify OSA severity as follows: mild (5–15 events/hour), moderate (15–30 events/hour), and severe (>30 events/hour). These thresholds are established by the American Academy of Sleep Medicine (AASM) and are widely utilized in clinical practice [2]) and associated with excessive daytime sleepiness (EDS) [2,3]. These events lead to cycles of hypoxemia and hypercapnia, increased sympathetic activity, and activation of the renin−angiotensin−aldosterone system, contributing to adverse metabolic and cardiovascular effects [4]. Polysomnography (PSG) remains the gold standard for diagnosis [5], but its limited accessibility has led to increased use of portable diagnostic tools in both research and clinical settings [5,6,7].

With a highly variable prevalence depending on definitions and diagnostic criteria, it is estimated that 1 in 5 adults have at least mild OSA, while 1 in 15 have moderate to severe forms [8]. In earlier reports, OSA was estimated to affect 2–5% of adults and up to 20% of the elderly population (>70 years) [6]. However, more recent data from the HypnoLaus study report substantially higher prevalence, with moderate-to-severe OSA affecting ~50% of men and ~20% of women [9]. OSA prevalence increases with age, body mass index (BMI), and upper airway abnormalities [10,11,12]. Despite its high prevalence, OSA is still under-recognized and underdiagnosed. Studies estimate that 50–90% of cases, particularly moderate-to-severe forms, may go undetected, especially in low-resource settings and among high-risk populations such as atrial fibrillation (AF) patients, who often lack symptoms like daytime sleepiness [6,13,14,15].

Untreated OSA is associated with increased cardiovascular risk, including hypertension, heart failure (HF), stroke, coronary artery disease (CAD), pulmonary hypertension, and arrhythmias [5,16,17,18,19,20,21,22]. It also significantly reduces life expectancy—by as much as 20 years in severe untreated cases [7]. OSA significantly affects perioperative outcomes and is associated with increased postoperative complications, including readmissions and new-onset AF, particularly after cardiac surgery [23,24].

AF is the most common sustained arrhythmia worldwide, particularly in older adults—more than one-third of patients are aged ≥80 years [25,26]. The global incidence continues to rise, contributing significantly to the socioeconomic burden on healthcare systems [25,27]. In 2010, worldwide prevalence was estimated at 596.2 per 100,000 men and 373.1 per 100,000 women, with projections suggesting a twofold increase by 2060 in the European population aged ≥55 years [27]. Notably, AF is associated with a fivefold increased risk of stroke and a twofold increased risk of cardiac death [26]. AF is also the most common arrhythmia found in patients with OSA [16,28], and the two conditions share several risk factors, including hypertension, diabetes, obesity, and structural heart disease [26,29].

Several studies have demonstrated a strong bidirectional relationship between OSA and AF [14,30,31]. The mechanisms underlying this association are complex and multifactorial and may involve oxidative stress, inflammation, autonomic dysfunction and imbalance, and atrial remodelling [24,31,32,33]. These pathophysiological changes may contribute to the initiation and worsening of AF in patients with OSA.

The prevalence of OSA in AF patients varies considerably, with reports ranging between 21 and 82% [14,16,25,34,35]. A recent study using home sleep apnea testing (HSAT) found an OSA prevalence as high as 90% in patients with non-valvular AF [36]. Approximately 40–50% of patients with AF have coexisting OSA [30,37], and the prevalence of AF increases fourfold in patients with severe OSA [16].

OSA adversely affects AF treatment outcomes, including response to antiarrhythmic drugs [38], recurrence after ablation [39,40], and electrical cardioversion [41]. It is also associated with an increased incidence of postoperative AF following coronary artery bypass grafting (CABG) [24]. While observational studies and meta-analyses support a beneficial effect of continuous positive airway pressure (CPAP) therapy on reducing AF recurrence [42,43,44], recent randomized controlled trials have challenged the protective effect of CPAP, highlighting the need for further research [45].

Given this strong interconnection, current guidelines continue to recommend OSA screening in patients with AF, particularly in those with resistant hypertension, obesity or HF, as managing OSA may improve arrhythmia and overall outcomes [26,29,33]. These recommendations are grounded in the evidence that arrhythmias such as AF and non-sustained ventricular tachycardia are significantly more likely to occur after a respiratory event than after normal breathing, as shown in analyses from the Sleep Heart Health Study and the DREAM study [16,28,35].

Taken together, these findings underscore the critical importance of early and accurate OSA diagnosis in patients with or at risk for AF. Timely recognition may support prevention strategies, inform personalized management, and ultimately improve patient outcomes. In this context, the present systematic review aims to synthesize the most recent data on the prevalence and diagnostic approaches of OSA in AF, providing comprehensive evidence to inform clinical practice and guide future research.

## 2. Methods

A systematic review was conducted in peer-reviewed databases, including PubMed and Google Scholar, to obtain cohort studies and clinical trials published in English, Portuguese or Spanish between 1 January 2019 and 9 December 2024.

The literature search was performed using the following keywords: (“Atrial Fibrillation” OR “Paroxysmal Atrial Fibrillation” OR “Fibrillation”) AND (“Sleep Apnea” OR “Obstructive Sleep Apnea” OR “OSA”). The results were analyzed by two independent reviewers. Non-concordances were resolved by consensus.

Data on the selected studies were synthesized into a data extraction sheet developed to comprise relevant information needed for further analysis and sections of this review. The topics of analysis included general study characteristics and the reported prevalence of SDB in AF populations; clinical presentation, including self-reported symptoms and structured assessment tools (e.g., STOP-Bang, ESS, Berlin questionnaire); diagnostic and screening methods for SDB; and echocardiographic, polysomnographic and laboratory findings.

This systematic review was conducted according to the PRISMA guidelines 2020 (Table S1 and S2).

## 3. Results

### 3.1. Search Results

**Figure 1:**
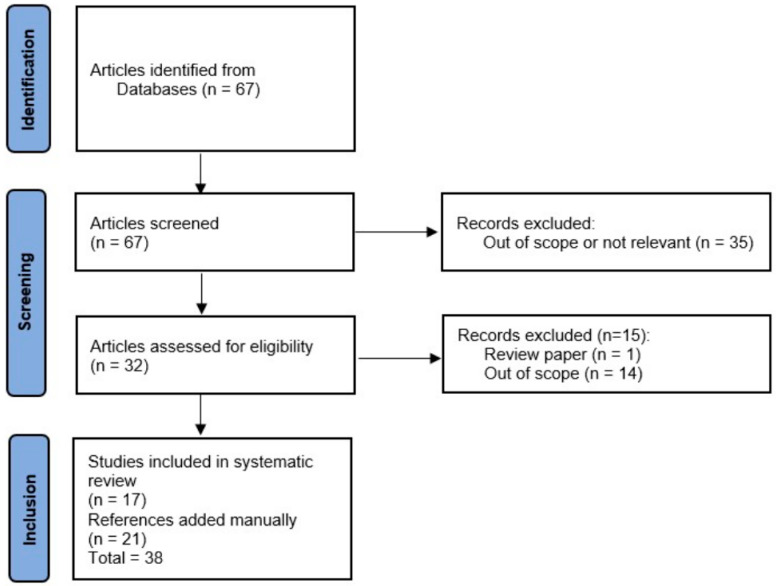
PRISMA flowchart.

The search strategy retrieved a total of 67 studies and the PRISMA flowchart of the study selection is presented in Figure 1. Forty-nine papers were excluded because they were out of scope or not relevant for this review and one because it was a review article. Twenty-one additional references detected in crosschecking searches and reference lists were manually added by the authors due to their significance for the topic of interest. In total, 38 eligible articles were included for qualitative evaluation.

### 3.2. Study Characteristics

The temporal distribution of the included studies was as follows: 2019 (*n* = 6), 2020 (*n* = 10), 2021 (*n* = 3), 2022 (*n* = 5), 2023 (*n* = 10), and 2024 (*n* = 4). Sixteen studies were conducted in Europe, followed by eleven studies from the United States. Additional countries represented included Australia (*n* = 3), China (*n* = 2), Japan (*n* = 2), South Korea, Tunisia, Turkey, and Canada. A detailed summary of the included studies is provided in Table 1.

With respect to study design, the majority were observational studies (*n* = 34), while a minority were randomized controlled trials (*n* = 4). Among the observational studies, seventeen were prospective studies, six were retrospective analyses, and seven were cross-sectional studies. Additionally, two studies were classified as quality improvement projects, one focused on the development and validation of a diagnostic model, and one was a matched case–control study.

Sample sizes varied considerably across studies, ranging from small (≤100 patients) [46,59,70] to moderate-sized cohorts (101–500 patients) [60,62,79], and large-scale studies (>500 patients) [23,52,58,66].

### 3.3. Study Populations

Most studies included adult patients with a diagnosis of AF, primarily middle-aged to elderly individuals, typically aged between 60 and 75 years. Across the included studies, the mean age of patients was 63.7 years (95% CI, 61.5–65.9 years; range 49.9–78.0 years), with a mean proportion of male participants of 63.2% (95% CI, 59.8–66.7%; range 45.0–80.1%). These participants were commonly recruited from inpatient cardiology units, outpatient clinics, or during assessments for catheter ablation procedures.

The study populations were diverse and included patients undergoing cardiac surgery—such as CABG or valve replacement—with or without AF, individuals with new-onset AF [50], and those scheduled for ablation procedures [54,67]. Several studies also involved patients with cardiac implantable electronic devices capable of monitoring SDB [7,48].

While some studies specifically excluded individuals with a prior diagnosis of OSA [54,67,71], others enrolled patients with OSA confirmed by PSG or identified through validated screening methods [59,60].

Furthermore, specific subgroups were represented, including elderly patients (e.g., Mehawej 2022; mean age ~75 years) [63] and individuals with heart failure with preserved ejection fraction (HFpEF) [79].

### 3.4. Methods of Sleep-Disordered Breathing Assessment Across Studies

There was substantial heterogeneity in the methods used to screen, diagnose, and monitor SDB across the included studies (Table 1). The gold standard in-laboratory PSG was used in several studies for definitive diagnosis [7,15,36,46,59] either alone or alongside other modalities.

Alternative diagnostic approaches included HSAT using devices such as WatchPAT [54,71,74,75], ApneaLink [55], and NightOwl [69], as well as respiratory polygraphy [56,76] and Type 3 sleep studies [47,61].

Some studies specified the duration of the sleep assessment. A single-night study was conducted in several HSAT protocols [54,65], while others extended monitoring to two nights, such as in Traaen 2020 and Hunt 2024 [57,76]. Jensen 2023 [69] used a four-night HSAT protocol with NightOwl, and Linz 2019 [48] and Mazza 2020 [53] used continuous night-to-night SDB tracking via pacemaker-based systems.

In a subset of studies involving cardiac device recipients, pacemaker-based algorithms such as the sleep apnea monitoring algorithm [48,52] or ApneaScan [53] were employed to estimate respiratory indices via transthoracic impedance, allowing for long-term or continuous SDB monitoring. Few studies relied on retrospective data sources such as clinical documentation, International Classification of Diseases (ICD) codes, or physician-reported diagnosis for the identification of SDB [23,49,51,73].

In terms of screening tools, the STOP-Bang questionnaire was the most frequently used tool [63,64,70], often combined with other instruments such as the Berlin questionnaire, Epworth Sleepiness Scale (ESS), or novel models like NABS (Neck circumference, Age, BMI, and Snoring) [15] and BOSS-GAP [67].

### 3.5. Prevalence of Sleep-Disordered Breathing in Atrial Fibrillation Populations

A total of 31 studies reported the prevalence of SDB conditions in patients with AF, as summarized in Table 2. Of these, 21 studies reported on OSA, seven reported on sleep apnea (SA) without specifying the type, and four assessed SDB more broadly.

Some studies included only patients with a confirmed or assumed diagnosis of SDB, thus not contributing to general prevalence estimates in unselected AF populations. In other studies, all participants had SDB by design or selection [46,72,77,79]. Vermeer’s 2023 study is ongoing and prevalence data has not yet been reported [75]. Two studies employed risk stratification tools without diagnostic confirmation: Oster 2022 [64] found that 53% of patients were at moderate to high risk for OSA (STOP-Bang score ≥ 3), while Rogel 2024 [78] reported that 25% were at high risk (STOP-Bang score ≥ 5).

Prevalence estimates ranged from 5% to 90%, depending on the specific condition assessed, diagnostic method used (e.g., PSG, screening questionnaires, or device-based monitoring), patient population, and severity thresholds. Despite methodological variability, most studies reporting OSA prevalence found rates above ~60%, underscoring the substantial burden of OSA in AF populations.

### 3.6. Severity Stratification of Sleep-Disordered Breathing in Atrial Fibrillation Populations

Among studies reporting OSA severity in AF populations, mild cases ranged from 23% to 75%, moderate from 18% to 50%, and severe from 7% to 48%. Despite some heterogeneity, a consistent pattern of moderate-to-severe disease emerged across studies. Several studies reported relatively balanced distributions across severity categories, suggesting a uniform and substantial disease burden [7,36,46,50,68,70]. Others identified higher proportions of severe OSA (approximately 33–48%), particularly among patients with persistent AF or comorbidities like HFpEF [58,59,61,79]. In contrast, some studies reported a predominance of mild OSA and relatively fewer severe cases [55,60,62,71]. Hunt 2024 found moderate-to-severe OSA in 42% of patients, underscoring its clinical relevance in AF populations [76].

In studies that reported SA without specifying the obstructive type, similar severity patterns were seen. Predominance of mild cases varied, accounting for nearly 44%, moderate cases had a prevalence between 33% and 51%, while severe OSA was observed in 12–31% of cases [15,54,57,69,74]. Moderate-to-severe SA was observed in approximately 42–56% of patients [15,57,69]. Higher proportions of severe SA were reported by Mazza 2020 (~80% in pacemaker recipients), highlighting a substantial burden in selected populations [53].

Among studies reporting SDB, Kadhim 2024 [77] identified SDB in approximately 66–86% of patients, and significant SDB (AHI ≥ 15) in approximately 34–54%, while Verhaert 2022 [65] and Betz 2023 [67] reported moderate-to-severe SDB in approximately 51–55%, with ~17% classified as severe. Linz 2019 [48], focusing specifically on device-based detection, found severe SDB in ~32% of patients using pacemaker-derived monitoring [48].

### 3.7. Prevalence of Atrial Fibrillation in Populations with Sleep-Related Breathing Disorders

Twelve studies referred to the prevalence of AF among patients with OSA, with reported rates ranging from 2% to 74%, depending on population characteristics and OSA severity. In general, AF prevalence was relatively low in population-based cohorts, ranging from approximately 2.5% in Blanchard 2021 [58] to 7.3% in Højager 2022 [61]. Higher prevalences were observed in clinical or selected populations, varying between approximately 18% in Rogel 2024 [78] and 37% in Rivas 2023 [73]. Feng 2019 [23] reported an AF prevalence of approximately 40% in postoperative cardiac surgery patients, while Gonçalves 2019 [7] documented a 42% AF rate during follow-up in pacemaker recipients. Despite earlier evidence suggesting that OSA increases the risk of AF recurrence following ablation, Hojo 2019 [47], focusing on recurrence after pulmonary vein isolation (PVI) in patients with pre-existing AF, found no significant association [47].

Højager 2022, Holtstrand 2023, and Wei 2024 reported a clear association between increasing OSA severity and higher AF prevalence [61,68,79].

### 3.8. Risk Factors for Sleep-Disordered Breathing in Patients with Atrial Fibrillation

The clinical characteristics that distinguish patient populations across the included studies are summarized in Table 3.

Frequently reported and consistent risk factors for SDB in patients with AF included demographic factors, namely male sex [23,45,50,51,52,55,56,58,61,62,63,68,71,74], and older age [45,47,54,56,61,62,67,68,69,70,71], although two studies found more prevalent OSA among younger patients [23,66].

Anthropometric factors such as higher BMI or obesity [23,47,49,50,54,55,57,58,60,61,62,63,65,67,68,69,70,71,79], as well as larger neck circumference [55,60,69] were also identified as significant risk factors.

Cardiometabolic comorbidities, such as hypertension [23,47,50,54,55,58,60,61,63,65,67,68,69], diabetes mellitus [23,47,51,58,61,62,63,77], history of thromboembolic events [67,68,69,77], cardiovascular disease [51,52,65,68], renal disease or failure [23,63], dyslipidemia, metabolic syndrome, and elevated HbA1c were also reported as relevant factors associated with OSA [61,67].

AF-related characteristics were also commonly associated with OSA [36,60,62,69,71,77]. Longer AF duration (greater than two years) was associated with OSA [36,57], as a higher CHA_2_DS_2_-VASc score [50,60,65,67,69,71,77].

Echocardiographic findings included increased left atrial size (diameter or volume) in several studies [15,60,77,79]. In contrast, Latif 2022 reported a smaller left atrial diameter in SA patients [62]. Regarding left ventricular ejection fraction (LVEF), results were mixed: Latif 2022 [62] found a lower LVEF in SA patients, while Jensen 2023 [69] found a higher LVEF among those with moderate-to-severe SA. In contrast, Hojo 2019 and Hunt 2024 found no significant differences in LVEF between OSA and non-OSA groups [47,76]. Additionally, Wei 2024 reported other structural cardiac changes associated with OSAHS and AF, including increased dimensions of the right ventricle, right atrium, and the right ventricular outflow tract [79].

### 3.9. Symptom Evaluation Scores for Sleep-Disordered Breathing

The ESS was extensively used to assess daytime sleepiness [7,15,36,50,55,57,58,60,61,68,71,74,76,77]. Mean ESS scores ranged from 5.5 to 11 across studies. While most studies did not find significant differences between groups, Holtstrand, 2023 [68] observed increasing ESS scores with greater OSA severity (Table 3).

The STOP-Bang questionnaire was also a commonly used tool to screen for SDB risk [15,50,54,55,57,60,64,68,71], with several studies reporting an increasing proportion of positive STOP-Bang results across OSA, SA or SDB severity levels [55,57,60,67,71].

The Berlin Questionnaire was used less consistently [36,50,57,60]. In two studies, more frequent high-risk classifications were found for OSA patients [57,60]. In addition, Starkey 2020 stratified symptom scores—such as STOP-Bang, ESS, NoSAS (Neck, Obesity, Snoring, Age, Sex), and snoring—across OSA severity categories [55]. Although no statistical comparisons were reported, the data showed an apparent upward trend in STOP-Bang and NoSAS scores with increasing OSA severity, as well as higher snoring prevalence in OSA groups.

Some studies did not report any data on self-reported symptoms related to OSA, SA, or SDB [59,62,65,75,79].

### 3.10. Self-Reported Symptoms of Sleep-Disordered Breathing

Snoring was one of the most reported symptoms across studies, with prevalence typically ranging from 60% to over 90% in patients with SDB and increasing with disease severity (Table 3). This symptom was assessed either by direct patient questioning or structured questionnaires. In several studies, snoring prevalence differed significantly between groups. Snoring was reported in 40–90% of OSA patients versus 0–60% of non-OSA [36,60,67]. By contrast, Gonçalves 2019 and Lin 2023 found no statistical differences in snoring between OSA patients and non-OSA patients [7,70].

Witnessed apneas, often reported by bed partners or via questionnaires, were documented in several studies [7,54,57,64,67,70]. The reported rates of observed apneas varied across studies, but moderate-to-severe SDB groups showed a higher prevalence compared to controls or those with mild disease. Traaen 2020, observed that apneas were present in approximately 12% (no SA), 18% (mild SA), 37% (moderate SA), and 44% of cases (severe SA), with *p* < 0.001 [57]. In Betz 2023, 34% of patients with moderate-to-severe SDB reported witnessed apneas, compared to only 10–17% in mild or no SDB (*p* = 0.003) [58] By contrast, three studies found no significant differences between groups [7,54,70].

General symptoms such as tiredness, fatigue, or EDS were reported differently across studies. Most studies assessed EDS using the ESS, while others evaluated fatigue through symptom checklists or other questionnaires. Among studies using ESS, higher scores were observed in patients with OSA, SA or SDB, but these differences were not statistically significant [36,60,77]. In Holtstrand 2023, although mean ESS scores increased significantly with OSA severity (*p* < 0.001), the proportion of patients with EDS (ESS ≥ 11) remained similar across groups, and no *p*-value was reported for that comparison [68]. In parallel, fatigue or tiredness was also reported based on patient self-report, without statistical significance between groups [36,54,70]. In Oster 2022, 71% of patients in the intervention group reported fatigue, though no control comparison was available [64].

### 3.11. Laboratory, ECG, Echocardiographic and Polysomnographic Findings

AHI, Respiratory Disturbance Index (RDI), or peripheral AHI (pAHI) were the most consistently reported objective markers across studies (Table 4) [15,36,47,50,52,53,56,58,60,61,62,65,68,69,70,71,72,76,77,80]. As expected, these indices were significantly higher in patients with OSA or with greater disease severity [47,60,68,69,77]. These indices were often stratified by clinical or demographic variables such as sex, BMI/obesity, hypertension status, or pacemaker-derived respiratory data [50,52,53].

The Oxygen Desaturation Index (ODI), measured at 3% or 4% thresholds, was another commonly used marker of SDB severity. Not surprisingly, ODI values were found to be higher in patients with SDB [58,60,65,68,69,71,76].

Oxygenation metrics, particularly those derived from peripheral oxygen saturation (SpO_2_), were often used to characterize the severity of nocturnal hypoxemia across studies. These included nadir SpO_2_, mean SpO_2_, and time spent below 90% saturation. Minimum SpO_2_ values were consistently lower in patients with SDB, varying between 79 and 81% in OSA vs. 84–92% in non-OSA [58,65,69]. In Holtstrand 2023, mean SpO_2_ also declined progressively with increasing OSA severity (95.9% in no OSA to 93.0% in severe OSA, *p* < 0.001) [68]. In addition, several studies assessed the cumulative burden of hypoxemia. Mills 2023 reported that time spent with SpO_2_ < 90% increased from 0.03 min in patients without OSA to 8.16 min in those with moderate-to-severe OSA [71]. Verhaert 2022 found a median time < 90% of 0 min in none/mild SDB and 7.0 min in severe SDB (*p* < 0.01) [65]. In Blanchard 2021, the proportion of time with SpO_2_ < 90% rose from 1% in patients without AF to 4% in those with incident AF (*p* < 0.0001) [58].

Biomarkers were less reported across the included studies and showed variable associations with SDB. B-type natriuretic peptide (BNP) and N-terminal pro-BNP were evaluated in Hojo 2019, Hunt 2024, and Wei 2024 [47,76,79]. Among these, only Wei 2024 demonstrated a significant stepwise increase in BNP levels corresponding with AF severity in OSAHS patients [79]. In contrast, Hojo 2019 reported no significant differences in BNP between non- and untreated OSA groups [47]. Creatinine levels were also assessed, with inconsistent findings [47,70,76].

Hemoglobin-related measures were reported in Mehawej 2022, Højager 2022, and Hunt 2024 [61,63,76]. Total hemoglobin was significantly higher in high-risk OSA patients based on STOP-Bang [63] and a progressive increase in HbA1c levels with increasing OSA severity was reported [61].

Also, visfatin, an inflammatory marker, was evaluated in Szymańska 2020, showing a positive correlation between plasma concentration and OSA severity [56].

Autonomic function measures, though rarely reported across the included studies, were detailed in Blanchard 2021 [58]. This study assessed heart rate variability parameters derived from nocturnal oximetry and found significant alterations in patients with incident AF and OSA. Specifically, those with incident AF had higher root mean square of successive differences and standard deviation of normal-to-normal intervals, showing increased overall variability, and a lower low-frequency to high-frequency ratio, suggesting parasympathetic predominance or reduced sympathetic modulation.

## 4. Discussion

### 4.1. Overview

Globally, an estimated 730 million individuals are affected by OSA [81]. Despite its high global burden, OSA is often an underrecognized condition, presenting with or without symptoms, and associated with substantial cardiovascular and neurocognitive morbidity [82]. OSA is characterized by recurrent episodes of nocturnal hypoxemia, hypercapnia, and autonomic activation, triggering systemic inflammation and oxidative stress. These mechanisms contribute to atrial remodelling and fibrosis, providing a pathophysiological substrate for the development and persistence of AF [83].

This systematic review synthesizes evidence on the prevalence and diagnosis of OSA in AF patients. Our review confirms the high prevalence of OSA in AF populations but also shows substantial methodological heterogeneity. This is reflected in the wide range of reported OSA prevalence, highlighting the need for consistent definitions and standardized diagnostic strategies.

### 4.2. Heterogeneity in Study Populations and Methodological Designs

Based on 38 studies published between 2019 and 2024, this review captures a growing global interest in the AF–OSA connection and the emerging diagnostic challenges in cardiology and sleep medicine, with significant contributions from Europe, North America, and Asia, and a predominance of observational studies. Sample sizes varied across studies, ranging from small cohorts to large-scale studies. Smaller studies typically focused on validating screening tools, while larger ones examined risk factors, outcomes, or prevalence of SDB and AF on a broader scale. Most studies included middle-aged to elderly AF patients (mean age ~64 years, ~63% male), often recruited during cardiology evaluations or ablation workups. Study populations were heterogeneous, including patients undergoing cardiac surgery, device recipients, and subgroups such as the elderly or those with HFpEF, which contributed to variability in the findings.

### 4.3. Diagnostic Approaches and Screening Tools

Studies included in this review employed a range of diagnostic methods, from gold-standard PSG to simplified ambulatory sleep studies, device-based monitoring, and screening questionnaires. Some studies relied on ICD codes or clinical diagnoses without formal testing, likely capturing only severe cases and introducing misclassification bias [84].

Screening tools such as STOP-Bang were widely used across studies, often alongside the ESS and the Berlin questionnaire. Although inherently subjective and lacking specificity when used alone, they offer practical value in busy clinical settings, but their accuracy is limited, particularly in minimally symptomatic AF patients [70,77]. Nonetheless, their use in systematic screening approaches may help find high-risk individuals for further evaluation.

Many studies relied on HSAT devices like WatchPAT or Level 3 monitors, rather than full PSG, the diagnostic gold standard. While HSAT offers advantages in cost and accessibility [85], it may lack accuracy in patients with comorbidities such as AF and may underestimate disease severity compared to PSG [74]. Inadequate stratification by severity and inconsistent AHI thresholds further contribute to this variability [86,87,88,89]. There was also a marked heterogeneity in the duration of sleep monitoring —ranging from a single night to multiple-night recordings. This variability likely affects diagnostic yield and may account for some of the inconsistencies in prevalence estimates. Linz et al. (2019) [48] proved that sleep apnea severity can vary significantly from night to night, suggesting that single-night testing may underestimate disease burden in some patients. Sousa et al., 2020 [90] emphasized the importance of considering how many nights should be assessed, as OSA severity fluctuates nightly [70,77].

These factors contribute to the broad range of reported prevalence estimates and highlight the importance of using standardized and sufficiently robust diagnostic and screening protocols in AF populations to ensure consistent classification, improve comparability across studies, and guide appropriate treatment decisions. Device-based monitoring, including pacemaker-derived respiratory data, represents a promising diagnostic avenue, particularly in patients already implanted with cardiac rhythm devices.

### 4.4. Underdiagnosis of OSA in AF Populations

A significant finding is the underdiagnosis of OSA in AF patients, which has been previously reported by other authors [91]. This is multifactorial, stemming from a general lack of awareness, the frequent absence of classic OSA symptoms, such as excessive daytime sleepiness—a presentation often referred to as the “non-sleepy” clinical phenotype common among cardiovascular patients [1,14,92,93]—and the limitations of relying on subjective screening tools alone [14,80]. In line with this, Lin 2023 [70] identified three major barriers to diagnosis and treatment of OSA in AF patients: (1) the lack of standardized and clinically validated predictors of OSA; (2) the lack of screening tools tailored to cardiovascular patients; and (3) the insufficient implementation and acceptance of PSG, despite its status as the diagnostic gold standard.

Traditional diagnostic criteria may miss a large subset of patients with significant disease burden, as many individuals with AF have few or non-classic OSA symptoms of loud snoring or excessive daytime sleepiness, leading to missed opportunities for diagnosis and treatment. This low symptom burden also reduces clinical suspicion among physicians. Compounding this challenge, the absence of a standardized diagnostic algorithm tailored to AF populations contributes to substantial heterogeneity in clinical practice—as seen across the studies reviewed—and likely plays a key role in persistent underdiagnosis. These factors reinforce the importance of objective testing, even in asymptomatic individuals, and the urgent need to establish evidence-based diagnostic pathways for this patient group.

Common symptoms, including snoring, unrefreshing sleep, fatigue, nocturia, and witnessed apneas, were inconsistently reported. These symptoms often lacked diagnostic value in this population, making early recognition of OSA in AF patients particularly challenging [1,14,70,90].

Several studies confirm that structured questionnaires like STOP-Bang, the ESS and the Berlin questionnaire, perform poorly in this population, and that newer models—such as the NABS score—may offer better predictive accuracy [15,92]. Nevertheless, they can serve as useful first-line options when incorporated into structured diagnostic pathways and supported by objective assessment methods [14]. Sousa et al. 2023 [92] suggested incorporating the STOP-Bang score into electronic health record systems to automate risk stratification and prompt timely referrals.

Another critical yet underexplored factor contributing to underdiagnosis is the duration of diagnostic monitoring. Most home-based assessments are limited to a single night, despite evidence showing substantial night-to-night variability in SA severity, particularly in patients with cardiovascular disease [48,57,76]. While multi-night recordings or continuous monitoring may increase diagnostic accuracy, their feasibility remains limited in routine practice. Therefore, there is a pressing need to develop and validate diagnostic methods that are both accurate and practical, ideally capable of reliably detecting clinically significant OSA in a single-night test.

Moreover, the limited accuracy of HSAT in AF patients, combined with low physician awareness and reluctance to undergo full PSG, further contributes to underdiagnosis [14,70].

These barriers highlight the need for AF-specific screening tools and initiative-taking diagnostic strategies, even in asymptomatic individuals, and support a broader conclusion: no screening questionnaire has proven ideal in the general population—and even less so in AF patients, who tend to be minimally symptomatic.

### 4.5. Prevalence Estimates

Our results showed considerable variability, with prevalence rates ranging from as low as 2% in population-based samples to as high as 74% in clinical subgroups. Across the reviewed literature, the prevalence of OSA in patients with AF was remarkably high, often exceeding 60% in studies that used objective diagnostic criteria such as PSG. Reported rates ranged from ~5% to 90%, reflecting methodological heterogeneity. Still, findings are broadly consistent with prior estimates suggesting sleep apnea affects ~50% of AF patients [37].

However, several studies relied on retrospective data sources such as ICD codes, clinical documentation, or physician-reported diagnoses, which may introduce diagnostic variability and misclassification bias, particularly where formal sleep testing was not systematically applied. Additionally, some studies included only patients with a confirmed or presumed diagnosis of sleep-disordered breathing, limiting their utility for estimating prevalence in unselected AF populations. As a result, data on OSA prevalence in the general AF population remain limited, partly due to the retrospective nature of some studies and the focus on selected, often symptomatic cohorts.

A consistent observation across studies was the high burden of moderate-to-severe OSA, particularly in patients with persistent AF or those undergoing rhythm control interventions such as catheter ablation or cardiac surgery. For context, prior reviews in the general population have reported OSA prevalence ranging from 9% to 38%, rising to approximately 49% in individuals aged ≥ 65 years [12], underscoring the amplified burden of OSA among patients with AF.

The inverse relationship—AF prevalence in OSA populations—was also examined, albeit in fewer studies. Multiple studies demonstrated that increasing OSA burden is associated with a higher prevalence of AF, supporting the concept of a severity-dependent risk of atrial arrhythmogenesis. In fact, available evidence supports a positive correlation between OSA severity and AF risk, burden, progression, and recurrence post-ablation [27,31,94,95].

### 4.6. Risk Factors and Clinical Correlates

OSA and AF share common risk factors [96,97], and both result in a significant reduction in quality of life [82]. Consistent with the published literature, risk factors for SDB in AF patients included male sex, older age, higher BMI, and cardiometabolic comorbidities such as hypertension, diabetes, and metabolic syndrome [82,98,99,100,101]. In addition to general cardiometabolic risk factors, several studies reported AF-specific characteristics associated with SDB [102,103]. Longer AF duration—particularly over two years—and higher CHA_2_DS_2_-VASc scores were linked to increased likelihood of OSA, suggesting that arrhythmia burden may be a relevant clinical marker for targeted screening [36,104]. Echocardiographic findings also showed inconsistency across studies: while some reported larger left atrial dimensions in patients with OSA, others described variable results in LVEF and right heart measurements, such as right atrial or right ventricular enlargement [62,79]. These discrepancies underscore the need for standardized reporting and further investigation into structural cardiac correlates of SDB in AF populations.

### 4.7. Clinical and Research Implications

Given the high prevalence of OSA in AF patients and the potential for improved outcomes with treatment, the underdiagnosis of moderate-to-severe OSA has significant clinical consequences [105], including increased cardiovascular morbidity and mortality [106], and a higher risk of AF recurrence post-ablation [107].

Integrating structured pathways for sleep-disordered breathing screening in arrhythmia units is warranted. Sousa et al., 2023 [92] proposes a clinical pathway with multidisciplinary approach to implement SDB diagnosis in AF patients. Symptom-based screening limitations support the need for objective tests like PSG even in asymptomatic patients. A low threshold for objective testing, such as PSG, should be considered, even in the absence of sleepiness or snoring.

Current guidelines recommend routine OSA screening in patients with AF, particularly in those with resistant hypertension, obesity, or heart failure [33], as managing OSA may improve arrhythmia and overall outcomes [14]. Despite these recommendations, routine implementation remains inconsistent. Gupta 2022 supports the idea that all patients with AF [101] should be screened for OSA, regardless of the presence of other risk factors [101]. Given the high burden of undiagnosed OSA in AF and its adverse impact on arrhythmia recurrence and cardiovascular outcomes, structured screening and diagnostic protocols should be prioritized. Interdisciplinary models involving sleep and cardiology services may improve detection, access to therapy, and long-term outcomes.

### 4.8. Study Limitations

This review benefits from a comprehensive and up-to-date evidence base, encompassing 38 studies from diverse geographic regions. However, several limitations must be acknowledged.

The search strategy was limited to specific languages (English, Portuguese and Spanish) and selected databases, which might have excluded relevant studies and introduced bias.

Marked heterogeneity in study designs, diagnostic methods, patient populations and sample sizes precluded quantitative synthesis and limited the comparability of results, which may affect the interpretation of overall findings.

Considering specifically the diagnostic methods, the approaches ranged from PSG to home sleep tests, screening questionnaires, and device-based algorithms. These tools differ in diagnostic accuracy, which may influence the reported prevalence rates. Additionally, some studies included in the review used only screening tools (e.g., STOP-Bang) without confirmatory testing, which may introduce misclassification bias and reduce diagnostic certainty.

Potential publication bias may have influenced these findings. Most included studies were observational and cross-sectional, which limits conclusions about causality [25] or long-term outcomes of OSA in atrial fibrillation populations. In addition, many studies lacked detailed sex- and ethnicity-specific analyses, constraining the generalizability of results.

Although this was a systematic review, the absence of a meta-analysis prevents pooled prevalence estimates or subgroup analyses, which would have strengthened our results. However, the heterogeneity of settings and results did not allow this analysis.

Large, prospective studies using PSG-confirmed OSA definitions are needed to better delineate prevalence and clinical impact. Randomized controlled trials assessing the effect of OSA therapy on AF outcomes are also warranted. Furthermore, validated AF-specific screening models are essential to enhance diagnostic accuracy [100]. Marulanda-Londoño 2017 suggest exploring reciprocal screening strategies between AF and OSA [108].

### 4.9. Unexpected Findings and Research Gaps

Some studies included in this review reported higher OSA prevalence among younger AF patients. This finding, while unexpected, underscores the importance of considering OSA in younger individuals with AF, regardless of traditional risk factors.

The lack of a consistent association between EDS and OSA severity in some studies suggests that symptom-based screening alone may not be sufficient for identifying all AF patients with clinically significant OSA.

The observation that OSA did not affect AF recurrence post-ablation in some studies highlights the complex interplay between these conditions and the need for further research to elucidate the mechanisms involved, namely atrial arrhythmogenesis in OSA, focusing on structural, electrical, and autonomic properties of the atria [47,97,108].

Several key research gaps remain. Large, prospective studies using full PSG are needed to accurately define the epidemiology and clinical implications of OSA in AF populations [109]. Efforts should focus on developing and validating AF-specific screening tools to enhance early recognition and individualized risk stratification, improving the efficiency and accuracy of OSA diagnosis in this population. Finally, randomized controlled trials evaluating the impact of OSA diagnosis and treatment on AF outcomes, such as AF recurrence, and cardiovascular outcomes, such as stroke, and mortality, are essential to guide clinical practice [110]. Sousa et al., 2020 suggests a multidisciplinary work to provide a better understanding of the correlation between cardiac biomarkers and polysomnographic features [90].

Furthermore, although a few studies investigated biomarkers such as BNP, visfatin, hemoglobin, and creatinine, the findings were inconsistent and often underreported. This limited and heterogeneous use of biochemical and cardiac markers represents an additional research gap. Further studies should explore inflammatory, oxidative stress, neurohormonal, and fibrosis-related biomarkers to improve risk stratification and support more personalized diagnostic strategies in AF patients with suspected OSA [97,111,112].

### 4.10. Future Directions and Research Priorities

In summary, this review underscores the high prevalence and diagnostic challenges of OSA in AF patients. The variability in prevalence estimates highlights the need for standardized diagnostic approaches, including the development and validation of a diagnostic algorithm, with a validated screening tool and a confirmatory testing, particularly the use of PSG. Integrating routine OSA screening into AF management protocols is essential, especially in high-risk individuals, by applying uniform inclusion criteria such as comorbidity profiles, age ranges and gender. Further research should focus on conducting large-scale trials with stablished methodological guidelines specifically for prevalence or diagnostic studies in obstructive sleep apnea, similar to the PRISMA framework, to reduce the heterogeneity observed across study designs included in this review.

## 5. Conclusions

OSA is highly prevalent among patients with AF, yet it remains significantly underdiagnosed due to limited symptom presentation, low clinical suspicion, and suboptimal use of diagnostic tools. The current body of evidence consistently demonstrates that moderate-to-severe OSA is common in AF populations and may adversely impact arrhythmia burden, treatment success, and cardiovascular outcomes.

This systematic review confirms the substantial variability in prevalence estimates, driven by methodological differences across studies, including diagnostic criteria, assessment modalities, and population characteristics. Despite these differences, the findings strongly support the integration of structured and objective OSA screening into routine AF care, even for asymptomatic individuals. Screening tools such as STOP-Bang may be useful when embedded into electronic health records, but they must be complemented by definitive diagnostic testing—ideally polysomnography—to avoid missed diagnoses.

Given the shared risk factors and overlapping pathophysiological mechanisms between OSA and AF, identifying and managing OSA in AF patients may offer an opportunity to reduce arrhythmia recurrence, optimize cardiovascular care, and improve long-term prognosis. However, the lack of universally accepted AF-specific screening pathways, coupled with inconsistent evidence from recent randomized controlled trials, highlights the need for further research. Future studies should prioritize large, prospective cohorts using standardized PSG-based definitions and evaluate the impact of OSA treatment on hard outcomes, such as stroke, mortality, and AF recurrence.

Ultimately, multidisciplinary collaboration between cardiology and sleep medicine will be essential to overcome current diagnostic barriers, personalize management strategies, and address the persistent gaps in evidence that limit current practice.

## Figures and Tables

**Table 1:** Summary of the studies’ characteristics and OSA/SA/SDB and AF prevalence.

First Author, Year Country	Objectives	Study Design	Patients Characteristics (*N*, % Male, Mean Age [Unless Otherwise Reported])	Diagnostic and Monitoring Assessments	Reported Prevalence
Caples, 2019 [46] USA	To assess the impact of CPAP treatment on the recurrence of AF after direct current cardioversion in OSA pts	Randomized controlled trial	25 pts with OSA and AF: PAP, 12 pts, 58% male, 63.5 ± 7.9 yrs Control, 13 pts, 54% male, 64.6 ± 10.1 yrs	OSA diagnosis: Attended split-night PSG	Mild OSA: PAP, 31% Control, 25% Moderate OSA: PAP, 31% Control, 42% Severe OSA: PAP, 38% Control, 33%
Feng, 2019 [23] USA	To understand the effect of OSA on hospital readmission rates and post-operative AF in the cardiac surgical population	Observational retrospective cohort study	506,604 pts after CABG or valve surgery, 68.2% male, 66.2 ± 12.1 yrs	OSA diagnosis: Based on clinical documentation (hospital records and clinical databases) and ICD-9 coding	OSA, 6.4% Post-operative AF in OSA pts, 40.4%
Gonçalves, 2019 [7] Portugal	To evaluate the diagnostic utility and accuracy of a PM-incorporated respiratory monitoring algorithm and its interaction with AF	Single-centre, observational prospective diagnostic accuracy study	81 pts with pacemakers, 58% male, 73 ± 11 yrs	OSA diagnosis: PSG Pacemaker respiratory monitoring algorithm tested as screening tool	OSAS, 62% OSAS severity: Mild, 40% Moderate, 30% Severe, 30% AF during follow-up in OSAS pts, 42%
Hojo, 2019 [47] Japan	To evaluate the impact of OSA on AF recurrence after multiple pulmonary vein isolation (PVI) sessions using a contact force-sensing catheter (CF-C)	Non-randomized, observational prospective cohort study	100 AF pts Non-OSA, 66 pts, 66.7% male, 60.4 ± 12.2 yrs Treated OSA (CPAP), 11 pts, 72.7% male, 66.3 ± 9.6 yrs Untreated OSA, 23 pts, 82.6% male, 68.3 ± 8.8 yrs	OSA Diagnosis: AHI ≥ 15 (Type 3 sleep study or PSG in selected cases)	OSA, 34% AF recurrence, 1st session: Non-OSA, 18.2% OSA, 14.7% AF recurrence, 2nd session: Non-OSA, 12.1% Treated OSA, 9.1% Untreated OSA, 8.7%
Linz, 2019 [48] Australia	To investigate night-to-night variability in SDB severity and its relationship with the daily risk of incident AF using simultaneous long-term day-by-day SDB and AF monitoring	Observational prospective cohort study (VARIOSA-AF Study)	72 pts with dual-chamber pacemakers capable of monitoring nightly SDB and daily AF burden, sex and age not reported	SDB Monitoring: Pacemaker-based SAM algorithm (RDI via transthoracic impedance)	Severe SDB, 32%
Providência, 2019 [49] Europe (7 countries)	To assess the impact of BMI on AF ablation outcomes, with emphasis on OSA as an independent predictor	Non-randomized, multicentre, international, observational prospective cohort study	2497 consecutive AF pts undergoing CA for AF, 70.6% male, 61.1 ± 10.2 yrs	OSA Diagnosis: Clinical history and sleep specialist referral	OSA, 7.0%
Bazan, 2020 [50] Spain	To investigate the prevalence of previously undetected OSA in pts with new-onset AF and provide a practical screening strategy for CPAP indication	Observational prospective diagnostic study	73 pts with new-onset AF, 67% male, 61 ± 10 yrs	OSA Diagnosis: Ambulatory RP (ApneaLink, ResMed); AHI ≥ 5	OSA, 82% OSA severity: Mild, 26% Moderate, 26% Severe, 30%
Ben Halima, 2020 [36] Tunisia	To determine the prevalence and severity of OSAS in pts with NVAF and identify OSAS’s predictive factors in this population	Cross-sectional study	100 NVAF, 45% male, 66.4 ± 9.7 yrs	OSAS Diagnosis: PSG confirmation Screening Tools: Berlin Questionnaire and ESS	OSAS, 90% OSAS severity: Mild, 32% Moderate, 27% Severe, 31%
Gellert, 2020 [51] USA	To investigate the association between SA and AF, premature atrial contractions, and premature ventricular contractions using 48 h ambulatory ECG	Observational, retrospective population-based cohort study from the Atherosclerosis Risk in Communities (ARIC) study	1154 pts, middle-aged to older adults SA, 45% male Non-SA, 34% male	SA Diagnosis: ICD-9 codes or self-reported physician diagnosis	SA, 18.8% AF, 6%
Martí-Almor, 2020 [52] Europe	To evaluate the incidence and severity of SA and its association with AF in an unselected population after implantation with an SA monitoring–enabled dual-chamber pacemaker	Multicentre, international, open-label, observational prospective cohort study (RESPIRE study)	553 pts, 61.5% male, 72.2% aged 65–85 yrs	SA Severity: Pacemaker SAM algorithm (transthoracic impedance for respiratory disturbances)	Severe SA, 31.1% Significant AF at 12 months: 25.0% (severe SA) vs. 13.9% (non-severe SA), *p* = 0.002 Persistent AF at 12 months: 16.9% (severe SA) vs. 7.3% (non-severe SA), CI 2.5–16.6% (*p* not explicitly stated but significant)
May, 2020 [15] USA	To evaluate and optimize SA screening tools in AF pts and to develop a novel screening model	Case–control study with matched controls (sex, race, age, BMI) within the SAFEBEAT trial	300 pts, 63.3% male, 61.4 ± 11.9 yrs PAF, 150 pts Control, 150 pts	SA Diagnosis: Full overnight PSG with manual scoring by certified polysomnologists; AHI ≥ 5 and ≥15 used to define SA presence and severity Screening Tools: STOP, STOP-BANG, Berlin, NoSAS, and ESS; a novel model (NABS: neck circumference, age, BMI, snoring) was developed and validated	SA PAF group AHI ≥ 5, 68.0% AHI ≥ 15, 43.3% SA Control group AHI ≥ 5, 69.3% AHI ≥ 15, 46.0%
Mazza, 2020 [53] Italy	To investigate pacemaker-detected SA variability, assess its association with AF and evaluate the associated long-term risk of stroke and death	Observational prospective cohort study, using pacemaker algorithms to detect SDB	439 pacemaker recipients, 57% male, 78 ± 8 yrs	SA Monitoring: Pacemakers with ApneaScan algorithm (Boston Scientific) to detect SA via thoracic impedance changes	Severe SA, RDI ≥ 30 episodes/h, 80% AF ≥ 6 h, 29%
Shapira-Daniels, 2020 [54] USA	To assess the prevalence of undiagnosed SA in AF pts undergoing ablation and its impact on SA therapy adherence	Observational prospective study at two tertiary referral centres	188 AF pts scheduled for CA, without a prior diagnosis of SA, 65.4% male, 62 ± 11.3 yrs	OSA Diagnosis: HSAT (WatchPAT), single-night session	SA, 82.4% SA severity: Mild, 43.8% Moderate, 32.9% Severe, 23.2%
Starkey, 2020 [55] Canada	To compare the accuracy of different OSA screening tools (NoSAS, STOP-BANG, AP) with HSAT in an AF population	Observational prospective diagnostic accuracy study	188 pts with NVAF, 61.2% male, 69.0 ± 9.5 yrs	OSA Diagnosis: HSAT (ApneaLink, ResMed); manually scored by respiratory therapists and interpreted by a sleep physician	OSA, 86% OSA severity: Mild, 36.7% Moderate, 32.4% Severe, 17.0%
Szymańska, 2020 [56] Poland	To assess the association between visfatin concentrations and OSA in AF pts	Observational study	266 hospitalized pts with AF, 65%, 57.6 ± 10.1 yrs	OSA Diagnosis: Overnight respiratory polygraphy (Embletta MPR); manually scored per AASM guidelines by certified sleep specialist	OSA, 45.5%
Traaen, 2020 [57] Norway	To study the prevalence, characteristics, risk factors, and type of SA in ablation candidates with PAF	Observational prospective cohort study	579 pts with PAF, 72.9% male, 59.9 ± 9.6 yrs	SA Diagnosis: Home Type 3 polygraphy over two nights (NOX Medical device)	SA, 82.7% SA severity: Moderate–severe (AHI ≥ 15), 42.1% Severe (AHI ≥ 30), 12.1%
Blanchard, 2021 [58] France	To evaluate whether oxygen desaturation and PRV indices derived from nocturnal oximetry are associated with incident AF in pts investigated for OSA	Multicentre, observational prospective cohort study (Pays de la Loire Sleep Cohort)	7205 AF-free pts, 62.3% male, median age 60 (IQR 50–70) yrs	OSA Diagnosis: PSG (52%) and RP (48%) Hypoxemia Assessment: Pulse oximetry (ODI, T90, nadir SpO_2_) and PRV metrics	OSA, 86.7% OSA severity: Mild, 22.8% Moderate, 22.4% Severe 41.5% AF, 2.5%
Delesie, 2021 [59] Belgium	To test the performance of OSA screening questionnaires/scoring scales in AF pts referred for PSG	Multicentre, observational prospective diagnostic validation study	100 pts with known previous AF, referred for PSG, 73% male, 64.0 ± 8.7 yrs	OSA Diagnosis: PSG	OSA (AHI ≥ 15), 69% Severe OSA (AHI ≥ 30), 33%
Mohammadieh, 2021 [60] Australia	To evaluate the diagnostic accuracy of commonly used OSA screening tools in AF pts and assess the prevalence of undiagnosed OSA in a hospital-based AF cohort	Prospective diagnostic accuracy study comparing multiple screening tools with gold standard PSG	107 pts with ECG-documented AF, 65.4% male, 61.3 ± 11.7 yrs	OSA Diagnosis: In-laboratory PSG (gold standard) and Level 3 HSAT (ApneaLink Air, ResMed) Screening Tools: Berlin Questionnaire, STOP-BANG, ESS, Mallampati score, BMI, snoring	OSA, 62.6% OSA severity: Mild, 31.8% Moderate, 18.7% Severe, 12.1%
Højager, 2022 [61] Denmark	To investigate the prevalence of silent AF and associated risk factors in pts with OSA	Multicentre, prospective cross-sectional study, conducted in two centres (hospital and private ENT clinic)	303 pts investigated for OSA, 68.6% male, 56.4 ± 12.4 yrs No/mild, 65 pts Moderate/severe, 238 pts	OSA Diagnosis: One-night home Type 3 sleep study (NoxT3™, ResMed); AHI used to classify severity	OSA, 78.5% OSA severity: Moderate, 30.7% Severe, 47.9% AF overall, 7.3% AF per OSA severity Mild, 1.5% Moderate/severe, 8.8% Severe, 10.7%
Latif, 2022 [62] USA	To analyze the prevalence of SA in AF pts and assess the impact of widespread SA screening on AF progression	Observational retrospective cohort study	185 AF pts, 72% male, median age 64 (IQR 58–71) yrs	OSA Diagnosis: HST and PSG	OSA, 49% OSA severity: Mild, 75% Moderate, 18% Severe, 7%
Mehawej, 2022 [63] USA	To examine the associations of OSA with frailty, cognitive performance, and AF-related QoL among older adults with AF	Observational, cross-sectional study based on prospective cohort data (the SAGE-AF study)	970 AF pts, 51% male, 75 yrs, 680 pts without OSA Low risk, 179 pt Intermediate, 360 pts High risk, 141 pts	OSA Diagnosis: Risk assessed using STOP-BANG questionnaire (not confirmed by PSG or HSAT) OSA Classification: STOP-BANG scores	OSA, 29.9%
Oster, 2022 [64] USA	To implement and evaluate the effectiveness of an evidence-based OSA bundle (including screening, education, and referral) in a hospitalized high-risk AF population	Pre-Post Quality Improvement project, with retrospective comparison group, evaluating the impact of a 3-month OSA intervention bundle on OSA screening, sleep clinic referral and follow-up	101 hospitalized AF pts Comparison, 68 pts, 48.5% male, 73.0 ± 11.6 yrs Intervention, 33 pts, 51.6% male, 69.0 ± 10.4 yrs	OSA Diagnosis: Screening using the STOP-Bang questionnaire; follow-up diagnosis via PSG or HSAT in referred cases STOP-Bang ≥ 3 triggered education and referral to a sleep clinic; OSA confirmed through formal sleep testing	Moderate to high-risk for OSA (modified STOP-Bang score ≥ 3), 52.9%
Verhaert, 2022 [65] Netherlands	To implement and evaluate a virtual sleep-SDB management pathway for AF pts scheduled for ablation	Observational prospective, remote SA screening study (VIRTUAL-SAFARI)	119 pts,55% male, 65 ± 9.5 yrs	OSA Diagnosis: One-night home sleep apnea test using WatchPAT-ONE or WatchPAT 300; data reviewed and interpreted by sleep physicians	Moderate-to-severe SDB, 55% SDB severity: Mild (AHI 5 < 15), 30% Moderate (AHI 15 < 30), 38% Severe (AHI ≥ 30), 17%
Ahn, 2023 [66] South Korea	To investigate age-related differences in clinical features, recurrence of atrial tachyarrhythmia, and predictors after radiofrequency CA for AF	Multicentre, observational prospective cohort study (Korean Heart Rhythm Society Ablation Registry for AF KARA)	2799 AF pts, 73.5% male, 59.9 ± 9.6 yrs Group A (<60 yrs), 1269 pts, 79.2% male, 51.6 ± 6.7 yrs Group B (≥60 yrs), 1530 pts, 68.7% male, 66.8 ± 5.2 yrs	OSA Diagnosis: Clinical history; no formal sleep study protocol described	OSA, 4.6% OSA prevalence: Group A, 6.5% Group B, 3.0%
Betz, 2023 [67] Netherlands	To assess the accuracy of STOP-BANG in detecting SDB in AF pts and to develop an AF-specific screening tool (BOSS-GAP score) to improve pre-selection for SDB testing	Observational prospective diagnostic accuracy study (sub-study of ISOLATION cohort and registry)	206 symptomatic AF pts scheduled for CA, without known SDB, 58% male, median age 65 (IQR 58–70) yrs	SDB Diagnosis: Home sleep apnea test using WatchPAT-ONE or WatchPAT 300; results reviewed by a sleep physician; AHI ≥ 15 defined moderate-to-severe SDB Screening Tools: STOP-Bang questionnaire assessed; refined into AF-specific BOSS-GAP score for improved pre-selection	Moderate-to-severe SDB, 51% SDB severity: Mild, 34% Moderate, 34% Severe, 17%
Holtstrand, 2023 [68] Sweden	To explore the association of OSA severity with AF in a sleep clinic cohort stratified by EDS	Cross-sectional study	3814 pts, 65.9% male, 53.8 ± 12.8 yrs	OSA Diagnosis: Home Sleep Apnea Test (HSAT) using Embletta^®^ device; manually scored by two sleep physicians; AHI used to define OSA severity (≥5, ≥15, ≥30 events/h for mild, moderate, and severe OSA) Sleepiness Assessment: ESS; EDS defined as ESS ≥ 11	OSA, 90.3% OSA severity: Mild, 23.7% Moderate, 35% Severe, 31.6% Baseline AF, 5.3% AF prevalence: Non-OSA, 1.6% Mild OSA, 3.9% Moderate OSA, 5.2% Severe OSA, 7.6%
Jensen, 2023 [69] Denmark	To assess SA prevalence and severity in AF pts using a home-monitoring device (NightOwl™) and evaluate its predictive value against CRM	Cross-sectional study	126 AF pts without known SA, 67% male, median age 68 yrs (IQR 60–75)	SA Diagnosis: Four-night home monitoring using NightOwl™ device; AHI ≥ 15 used to define moderate-to-severe SA; validation in a subgroup with CRM Screening Tool: NightOwl™ device (photoplethysmography -based sensor with smartphone integration); positive predictive value validated against CRM	Moderate to severe SA (AHI > 15), 56% SA severity: Mild (AHI 5–15), 36% Moderate (AHI 15–30), 28% Severe (AHI > 30), 29%
Lin, 2023 [70] China	To evaluate the application of STOP-BANG in screening OSAHS in AF pts	Cross-sectional study	63 AF pts, 50.8% male, 62.5 ± 10.7 yrs	OSAHS Diagnosis: STOP-BANG questionnaire and full overnight PSG (Philips Alice 5/6 system); AHI ≥ 5 events/h used to confirm OSAHS, with severity classification Screening Tool: STOP-BANG questionnaire	OSAHS, 82.5% OSAHS severity: Mild, 32.7% Moderate, 34.6% Severe, 32.7%
Mills, 2023 [71] USA	To define the prevalence of undiagnosed OSA in consecutive ambulatory AF pts and evaluate its impact on AF-related QoL	Observational prospective, cohort study (single-centre phase IV registry)	38 consecutive ambulatory AF pts investigated for OSA with WatchPAT, 68.4% male, median age 58.3 (IQR 52.0–69.0) yrs	OSA Diagnosis: One-night home sleep test using WatchPAT-One or WatchPAT-300; interpreted by board-certified sleep medicine specialists; AHI ≥ 5 defined OSA, AHI ≥ 15 defined moderate-to-severe OSA Screening Tools: STOP-Bang questionnaire and ESS used alongside HST	OSA, 79% OSA severity: Mild, 42,1% Moderate/Severe, 36.8%
Ozkan, 2023 [72] Turkey	To investigate the relationship between the visceral adiposity index and AF development in OSAS pts	Observational retrospective study	207 pts with OSAS, 80.1% male, 49.9 ± 4.06 yrs AF group, 44 pts NSR group, 163 pts	OSAS Diagnosis: Based on AHI; severity defined as mild (5–15), moderate (15–30), or severe (>30)	AF, 21.3%
Rivas, 2023 [73] USA	To evaluate the association between OSA and postoperative AF and delirium after cardiac surgery in DECADE trial pts	Sub-analysis of a multicentre, randomized, double-blind, placebo-controlled clinical trial (DECADE trial)	590 pts, sex and age not re-ported	OSA Diagnosis: STOP-BANG questionnaire score >5 and/or preoperative diagnosis of OSA (ICD-9 codes)	OSA, 23% AF incidence, 37%
Tanaka, 2023 [74] Japan	To compare HSAT using Watch-PAT and PSG in AF pts undergoing CA	Single-centre, observational retrospective study	464 consecutive AF pts, 76.5% male, 64.9 ± 10.6 yrs	SA Diagnosis: Both WatchPAT (home sleep apnea test) and in-lab PSG; AHI ≥ 5 used for SA classification; PSG used to determine CPAP indication	SA, 88.6% SA severity: Mild, 18.3% Moderate, 50.9% Severe, 30.0%
Vermeer, 2023 [75] Netherlands	To evaluate the impact of integrated, nurse-led lifestyle intervention program including OSA screening on AF ablation outcomes	Prospective, 1:1 randomized, controlled, single-centre, open-label, investigator-initiated clinical trial (POP Trial)	150 pts with paroxysmal or persistent AF referred for 1st PVI, sex not yet reported, age range 18–75 yrs	OSA Diagnosis: HSAT using WatchPAT™ 300; OSA suspected if AHI ≥ 5/h	Prevalence data not yet reported (ongoing study)
Hunt, 2024 [76] Norway	To evaluate the impact of CPAP treatment on LA and LV remodelling in pts with OSA and PAF before and after CA	Randomized controlled trial (A3 trial)	108 pts with PAF and moderate-to-severe OSA, 76% male, 63 ± 7 yrs CPAP, 55 pts Standard care, 54 pts	OSA Diagnosis: Respiratory polygraphy over two nights; OSA defined as AHI ≥ 15 events/hour	Moderate/severe OSA, 42%
Kadhim, 2024 [77] Australia	To develop and validate a prediction model (MOODS-AF) to estimate the probability of moderate-to-severe SDB (AHI ≥ 15/h) in AF pts	Multicentre, prospective cohort study with external validation cohort	851 total pts Derivation cohort, 442 ambulatory AF pts undergoing PSG, 69.2% male, 60 ± 11 yrs Validation cohort, 409 pts, 75.8% male, 59 ± 10 yrs	SDB Diagnosis: In-lab PSG; moderate-to-severe SDB defined as AHI ≥ 15 events/h Screening Tool: MOODS score developed and validated as a simplified clinical prediction tool based on sex, BMI, diabetes, and prior stroke/TIA	Any SDB: Derivation cohort, 66.1% Validation cohort, 86% Significant SDB: Derivation cohort, 34% Validation cohort, 54%
Rogel, 2024 [78] USA	To screen and stratify OSA risk in adult pts using the STOP-BANG during clinic visits over 6 weeks. To increase provider referrals for sleep studies in high-risk identified pts (STOP-Bang ≥ 5) following questionnaire’s implementation	Quality Improvement Project (prospective, observational, comparative study focused on evaluating OSA systematic screening impact in a cardiology patient population)	963 pts Pre implementation, 535 pts, 48% male, 68.2 ± 13.3 yrs Implementation, 428 pts, 279 pts screened with STOP-Bang, 49% male, 68.8 ± 11.1 yrs	OSA Risk Assessment: STOP-BANG questionnaire administered in outpatient cardiology clinic	High risk for OSA, 25% AF prevalence: Pre implementation, 19% Implementation, 16%
Wei, 2024 [79] China	To explore cardiac structural and functional changes in OSAHS pts with HFpEF and AF	Observational retrospective study	336 OSAHS pts with HFpEF Group A (non-AF), 187 pts, 50.3% male, 74.9 ± 9.2 yrs Group B (AF history, no episodes), 56 pts, 55.4% male, 71.3 ± 11.7 yrs Group C (AF history and episodes), 93 pts, 57.0% male, 73.9 ± 10.4 yrs	OSAHS Diagnosis: In-lab PSG using Embletta X20; severity classified based on AHI: mild (5–15), moderate (15–30), severe (>30)	Mild OSA: Group A, 63.1% Group B, 42.9% Group C, 11.8% Moderate OSA: Group A, 27.3% Group B, 37.5% Group C, 45.2% Severe OSA: Group A, 9.6% Group B, 19.6% Group C, 41.7% AF prevalence: Mild OSA, 22.9% Moderate OSA, 55.3% Severe OSA, 74.0%.

Legend: AASM, American Academy of Sleep Medicine; AF, atrial fibrillation; AHI, apnea–hypopnea index; AP, acoustic pharyngometer; BMI, body mass index; CA, catheter ablation; CABG, coronary artery bypass grafting surgery; CPAP, continuous positive airway pressure; CRM, cardio-respiratory monitoring; EDS, excessive daytime sleepiness; ENT, ear, nose, and throat doctor; ESS, Epworth Sleepiness Scale; HFpEF, heart failure with preserved ejection fraction; HSAT, Home Sleep Apnea Test; HST, Home sleep test; ICD, International Classification of Diseases; IQR, interquartile range; LA, left atrial; LV, left ventricular; NoSAS, Neck, Obesity, Snoring, Age, Sex; NSR, normal sinus rhythm; NVAF, non-valvular AF; ODI, oxygen desaturation index; OSA, obstructive sleep apnea; OSAHS, obstructive sleep apnea–hypopnea syndrome; OSAS, obstructive sleep apnea syndrome; PAF, paroxysmal atrial fibrillation; PAP, positive airway pressure; PM, pacemaker; PRV, pulse rate variability; PSG, polysomnography; pts, patients; PVI, pulmonary vein isolation; QoL, quality of life; RDI, respiratory disturbance index; RP, respiratory polygraphy; SA, sleep apnea; SAM, sleep apnea monitoring; SDB, sleep-disordered breathing; SpO_2_, oxygen saturation; T90, time under 90% oxygen saturation; USA, United States of America; yrs, years.

**Table 2:** Summary of reported OSA/SA/SDB prevalence.

Very High Prevalence (≥80%)	High Prevalence (60–79%)	Moderate Prevalence (30–59%)	Low Prevalence (<30%)
OSA Prevalence			
Bazan 2020 [50], ~82%	Gonçalves 2019 [7], ~62%	Mehawej 2022 [63], ~30%	Ahn 2023 [66], ~5%
Lin 2023 [70], ~83%	Mohammadieh 2021 [60], ~63%	Hojo 2019 [47], ~34%	Feng 2019 [23], ~6%
Starkey 2020 [55], ~86%	Delesie 2021 [59], AHI ≥ 15: ~69%	Hunt 2024 [76], 42%	Providência 2019 [49], ~7%
Blanchard 2021 [58], ~87%	Højager, 2022 [61], ~79%	Szymanska, 2020 [56], ~46%	Gellert 2020 [51], ~19%
Ben Halima 2020 [36], ~90%	Mills 2023 [71], ~79%	Latif 2022 [62], ~49%	Rivas 2023 [73], 23%
Holtstrand 2023 [68], ~90%			
SA prevalence			
Mazza 2020 [53], ~80% severe SA		Marti-Almor 2020 [52], ~31% severe SA	
Shapira-Daniels 2020 [54], ~82%		May 2020 [15], AHI ≥ 15: ~45%	
Traaen 2020 [57], ~83%		Jensen, 2023 [69], 56% moderate to severe SA	
Tanaka 2023 [74], ~89%			
SDB prevalence			
		Linz 2019 [80], ~32% severe SDB	
		Kadhim 2024 [77], ~76% any SDB, 44% significant SDB	
		Betz 2023 [67], ~51% moderate to severe SDB	
		Verhaert 2022 [65], ~55%, moderate to severe SDB	

Legend: OSA, obstructive sleep apnea; SA, sleep apnea; SDB, sleep-disordered breathing.

**Table 3:** Summary of clinical characteristics distinguishing patient populations in the included studies.

First Author, Year	Risk Factors	Symptoms of OSA/SA/SDB in AF
Caples, 2019 [46]	Control vs. PAP (all pts had OSA): No significant differences between groups in age (64.6 vs. 63.5 yrs), sex (54% vs. 58% male), BMI (35.8 vs. 36.0 kg/m^2^), LVEF (57.3% vs. 58.1%), or LAVI (35.6 vs. 40.6 mL/m^2^), all *p* > 0.05	Control vs. PAP (all pts had OSA): Baseline ESS: 7.3 ± 3.3 vs. 4.8 ± 2.1, *p* = 0.04; follow-up ESS: 5.7 vs. 5.8, *p* = 0.17 Baseline FOSQ: 17.6 ± 1.6 vs. 19.1 ± 0.7, *p* = 0.01; follow-up FOSQ: 17.5 vs. 18.3, *p* = 0.26
Feng, 2019 [23]	Risk factors for OSA vs. non-OSA: Younger age, male sex, white race, hypertension (uncomplicated and complicated), chronic pulmonary disease, diabetes (uncomplicated and complicated), renal failure, obesity, and depression were more prevalent among OSA pts	OSA diagnosis based on ICD-9 codes; symptoms not assessed
Gonçalves, 2019 [7]	No significant differences between pts with and without OSAS	Snoring, witnessed apneas, restless sleep, and EDS were more frequent in OSAS, but differences were not statistically significant Snoring: 68% (OSAS) vs. 42% (non-OSAS), *p* = 0.14. Witnessed SA: 20% (OSAS) vs. 19% (non-OSAS), *p* = 0.45. Restless sleep: 28% (OSAS) vs. 32% (non-OSAS), *p* = 0.74 EDS (ESS > 10): 14% (OSAS) vs. 6.5% (non-OSAS), *p* = 0.31.
Hojo, 2019 [47]	Risk factors for OSA vs. non-OSA: Older age, higher BMI, higher AHI, hypertension, and diabetes were significantly associated with OSA	Symptoms were not assessed; OSA diagnosed via sleep study based on AHI ≥ 15
Linz, 2019 [48]	Not reported	Not reported
Providência, 2019 [49]	Risk factors for OSA: Higher BMI	Not reported
Bazan, 2020 [50]	Risk factors for OSA vs. non-OSA: Male sex, higher BMI, hypertension, higher CHA_2_DS_2_-VASc score	Snoring, observed apneas, fatigue, daytime sleepiness: Not individually reported STOP-BANG score: 4.1 ± 1.6 (OSA) vs. 2.6 ± 1.9 (non-OSA), *p* = 0.004 ESS: 8.4 ± 4 (OSA) vs. 9.3 ± 3 (non-OSA), *p* = 0.47 (NS) Berlin Questionnaire: 1.6 ± 0.9 (OSA) vs. 1.2 ± 1.1 (non-OSA), *p* = 0.12 (NS)
Ben Halima, 2020 [36]	Risk factors for OSAS vs. non-OSAS: Age > 61 yrs and AF duration > 2 yrs	Snoring: 90% (OSAS) vs. 60% (non-OSAS), *p* = 0.024 [Independent predictor: OR = 18.9 (95% CI 1.62–221.1), *p* = 0.019] ESS: 10.5 ± 4.1 (OSAS) vs. 9 ± 4.5 (non-OSAS), *p* = 0.2 (NS) ESS > 10: 56% (OSAS) vs. 30% (non-OSAS), *p* = 0.1 (NS) Berlin Questionnaire: Positive in 34% (OSAS) vs. 50% (non-OSAS), *p* = NS Other symptoms (e.g., sleep disorders, nocturia, daytime sleepiness, fatigue, memory, and cognitive impairment): Not significantly different
Gellert, 2020 [51]	Risk factors for SA vs. non-SA: Male sex, white race, current smoking, diabetes, CHD	SA was identified via self-reported physician diagnosis or ICD-9 codes; symptoms not assessed or compared
Martí-Almor, 2020 [52]	Risk factors for severe SA (RDI ≥ 20) and non-severe SA: Older age, male sex, AF history, CAD, cardiomyopathy, MI, HF, cardiac surgery history, smoking history, more frequent use of beta-blockers	Not reported
May, 2020 [15]	Risk factors for AF vs. non-AF (controls): Lower HR, larger LAV, and more frequent use of beta-blockers and calcium channel blockers	STOP-BANG Score: 3.5 ± 1.4 (PAF) vs. 3.4 ± 1.5 (controls), *p* = 0.91 Berlin Questionnaire: 1.6 ± 0.8 (PAF) vs. 1.6 ± 0.9 (controls), *p* > 0.99 NoSAS Score: 10.8 ± 4.1 (PAF) vs. 11.0 ± 4.0 (controls), *p* = 0.64 ESS: 7.5 ± 4.3 (PAF) vs. 8.3 ± 4.3 (controls), *p* = 0.12
Mazza, 2020 [53]	Predictors of AF among pts with SA: RDI max ≥ 63 ep/h and RDI mean ≥ 46 ep/h RDI burden ≥ 7% not significantly associated with AF Death/stroke risk higher with AF ≥ 6 h In pts without AF history, RDI max ≥ 63 predicted death/stroke	Not reported, SA diagnosed via pacemaker-derived RDI; symptoms not assessed
Shapira-Daniels, 2020 [54]	Risk factors for SA vs. non-SA: Older age, higher BMI, and hypertension	Sleep symptoms (snoring, daytime sleepiness, fatigue, apneas): 69.0% (SA) vs. 69.7% (non-SA), *p* = 1.00 (NS) STOP-BANG score: median 4 [IQR 3–5] (SA) vs. 3 [IQR 2–4.5] (non-SA), *p* = 0.003 STOP-BANG positive: 81.2% (SA) vs. 57.6% (non-SA), *p* = 0.01
Starkey, 2020 [55]	Risk factors for OSA: Male sex, higher BMI, larger neck circumference, smoking, hypertension, and more frequent use of beta-blockers	Average ESS score: 5.9 ± 3.9, range 0–19; ESS > 11: 9% OSA screening-related parameters (non-OSA vs. mild OSA vs. moderate OSA vs. severe OSA, no *p*-values reported): Snoring: 46.2% vs. 73.9% vs. 63.9% vs. 75.0% ESS: 5.2 ± 4.1 vs. 6.2 ± 4.0 vs. 6.1 ± 3.8 vs. 5.2 ± 3.4 NC: 35.2 ± 3.7 vs. 38.1 ± 3.7 vs. 39.3 ± 3.7 vs. 40.7 ± 4.4 cm MCA: 2.4 ± 0.7 vs. 2.4 ± 0.9 vs. 2.5 ± 1.1 vs. 2.2 ± 0.6 cm^2^ NoSAS score: 8.1 ± 3.4 vs. 9.6 ± 3.7 vs. 11.2 ± 3.6 vs. 12.6 ± 4.1 STOP-BANG score: 2.9 ± 1.4 vs. 3.4 ± 1.4 vs. 3.9 ± 1.3 vs. 4.3 ± 1.6
Szymańska, 2020 [56]	Risk factors for OSA: Male sex, older age, higher BMI, more often arterial hypertension, history of stroke/peripheral thromboembolism and vascular disease, visfatin concentration > 1.25 ng/mL, permanent AF	Not reported
Traaen, 2020 [57]	Risk factors for SA Male sex, older age, higher BMI, habitual snoring, and AF duration	Values per SA severity (non-SA vs. mild SA vs. moderate SA vs. severe SA): Snoring: 55.6% vs. 66.5% vs. 85.9% vs. 81.2%, *p* = 0.056 Observed Apneas: 12.1% vs. 17.6% vs. 37.3% vs. 43.8%, *p* < 0.001 ESS score: 6.9 ± 4.3 vs. 7.1 ± 4.0 vs. 7.5 ± 3.6 vs. 7.1 ± 4.2, *p* = 0.367 Berlin Questionnaire (score ≥ 2): 25.3% vs. 36.4% vs. 54.1% vs. 58.8%, *p* < 0.001 STOP-BANG (score ≥ 3): 35.9% vs. 62.8% vs. 83.1% vs. 84.6%, *p* < 0.001
Blanchard, 2021 [58]	Risk factors for AF vs. non-AF: Older age, male sex, higher BMI, diabetes, cardiac diseases, hypertension, more frequent use of beta-blockers and calcium channel blockers, greater PAP adherence, more severe nocturnal hypoxemia (higher T90), and altered autonomic regulation (increased RMSSD and SDNN; lower LF/HF ratio)	ESS: median 10 [IQR 6–13] (AF) vs. 10 [IQR 6–14] (non-AF), *p* = 0.2096 Fatigue and EDS not significantly different; no significant difference in classic symptoms
Delesie, 2021 [59]	Not reported	Not reported
Mohammadieh, 2021 [60]	Risk factors for OSA (AHI ≥ 15) vs. non-OSA (AHI < 15): Higher BMI, larger neck circumference, higher Modified Mallampati score, hypertension, congestive cardiac failure, higher CHA_2_DS_2_-VASc score, persistent/permanent AF, and larger left atrial area	Self-reported snoring: 78.8% (AHI ≥ 15) vs. 58.1% (AHI < 15), *p* = 0.039 ESS score: 6.9 ± 3.4 (AHI ≥ 15) vs. 5.7 ± 3.3 (AHI < 15), *p* = 0.079 (NS) STOP-BANG score: 4.7 ± 1.7 (AHI ≥ 15) vs. 3.0 ± 1.3 (AHI < 15), *p* < 0.001 Berlin questionnaire (high risk): 75.8% (AHI ≥ 15) vs. 25.7% (AHI < 15), *p* < 0.001
Højager, 2022 [61]	Risk factors for OSA severity (moderate/severe vs. mild OSA): Older age (moderate and severe OSA), male sex (severe OSA), higher BMI (moderate and severe OSA), hypertension (moderate and severe OSA), dysregulated hypertension (moderate and severe OSA), type 2 diabetes (severe OSA), prediabetes (severe OSA), higher HbA1c (severe OSA), higher waist/hip ratio (severe OSA), higher systolic blood pressure (moderate and severe OSA), higher diastolic blood pressure (severe OSA), higher heart rate (severe OSA), metabolic syndrome (moderate and severe OSA), higher central apneas (severe OSA)	ESS mean score: 9.9 ± 4.8 No significant differences in ESS across OSA severity (*p* = 0.73): AHI < 15 (mild OSA): 9.5 ± 4.1 AHI 15–30 (moderate OSA): 9.9 ± 4.9 AHI > 30 (severe OSA): 10.1 ± 5.1
Latif, 2022 [62]	Risk factors for OSA vs. non-SA: Older age, male sex, higher BMI, higher diabetes prevalence, lower CAD prevalence, lower stroke prevalence, more paroxysmal AF and less persistent AF at diagnosis, smaller left atrial diameter, and lower ejection fraction	Not reported
Mehawej, 2022 [63]	Risk factors for high-risk OSA vs. low-risk OSA: Male sex, higher BMI, hypertension, diabetes, renal disease, and frailty	STOP-BANG used for risk stratification; no symptoms reported separately Cognitive impairment (MoCA ≤ 23): 45.6% (intermediate-risk OSA) vs. 35.2% (low-risk) vs. 32.6% (high-risk), *p* < 0.01
Oster, 2022 [64]	Not reported	Intervention group: Snoring: 80.6% Daytime tiredness: 71.0% Observed apnea: 32.3% Neck (>40 cm): 45.2% STOP-Bang score: Modified STOP-Bang (comparison): 2.6 ± 0.8 Complete STOP-Bang (intervention): 4.7 ± 1.3
Verhaert, 2022 [65]	Risk factors for moderate-to-severe SDB vs. none/mild SDB: Higher BMI, higher CHA_2_DS_2_-VASc score, congestive heart failure, hypertension, higher number of cardiovascular drugs (≥4), and more frequent use of beta-blockers and RAS inhibitors	Not reported
Ahn, 2023 [66]	Risk factors for OSA: Age < 60 yrs	Not reported
Betz, 2023 [67]	Risk factors for moderate-to-severe SDB vs. none/mild SDB: Age > 50 yrs, higher BMI, higher CHA_2_DS_2_-VASc score, dyslipidemia, hypertension, previous thromboembolic events (stroke or TIA), vascular disease, vitamin K antagonists use	Snoring: 40% in moderate-to-severe SDB vs. 0–28% in mild or non-SDB (*p* = 0.012) Observed apneas: 34% in moderate-to-severe SDB vs. 10–17% in mild or non-SDB (*p* = 0.003)
Holtstrand, 2023 [68]	Risk factors for OSA: Higher age, male sex, obesity, current smoking, hypertension, CAD, and stroke Risk for prevalent AF: increased in severe OSA without EDS, OR 2.54, 95% CI 1.05–6.16, *p* = 0.039, independent of confounding factors	Mean ESS score: 10.6 ± 5.0 ESS score per OSA severity (non-OSA, mild, moderate, severe): 10.0 (6.0–14.0) vs. 10.0 (6.0–13.0) vs. 11.0 (7.0–15.0) vs. 11.0 (7.0–15.0), *p* < 0.001 EDS (ESS ≥ 11): 50.8% EDS per OSA severity (non-OSA, mild, moderate, severe): 172 (46.5%) vs. 421 (46.6%) vs. 707 (52.9%) vs. 566 (53.0%), *p*-value not reported
Jensen, 2023 [69]	Risk factors for moderate-to-severe SA vs. none/mild SA: Older age, BMI, and neck circumference; higher resting HR; permanent AF; higher CHA_2_DS_2_-VASc score; hypertension; history of thromboembolic events; higher LV ejection fraction; and more frequent use of benzodiazepines and steroids	Not reported
Lin, 2023 [70]	Risk factors for OSAHS vs. non-OSAHS: Older age, higher BMI, postmenopausal women, and more frequent use of statins	STOP-Bang questionnaire (all NS): Snoring: 64.7% (OSAHS) vs. 36.4% (non-OSAHS), *p* = 0.101 Daytime fatigue: 72.6% (OSAHS) vs. 45.5% (non-OSAHS), *p* = 0.151 Observed apnea: 21.6% (OSAHS) vs. 9.1% (non-OSAHS), *p* = 0.675
Mills, 2023 [71]	Risk factors for moderate–severe OSA vs. mild and non-OSA: Older age, male sex, white race, higher BMI and CHA_2_DS_2_-VASc score, persistent AF	Non-OSA, mild, and moderate-to-severe OSA, no *p*-values reported: ESS: 6.0 (4.5–9.0), 6.0 (4.5–8.5), 5.0 (3.0–7.0) STOP-BANG: 3.5 (2.3–4.0) vs. 5.0 (3.0–5.0) vs. 4.5 (4.0–5.0)
Ozkan, 2023 [72]	Risk factors for AF vs. non-AF (NSR): Higher VAI, PAP, and SII	Not reported
Rivas, 2023 [73]	OSA was not associated with AF	Not reported
Tanaka, 2023 [74]	Risk factors for OSA vs. non-OSA: Male sex, WP-AHI ≥ 18.1 and ESS ≥ 11	ESS score: 6.8 ± 4.5 (overall, no group comparison provided) ESS ≥ 11: independent predictor of CPAP indication
Vermeer, 2023 [75]	Not reported	Not reported. STOP-BANG or symptom scores (ESS, fatigue, etc.) not included; OSA suspected if AHI ≥ 5 on WatchPAT
Hunt, 2024 [76]	No significant differences in cardiac structure and function changes between pts with or without OSA	ESS score: 7.4 ± 3.2
Kadhim, 2024 [77]	Risk factors for moderate-to-severe SDB vs. no/mild SDB: Male sex, higher body weight and BMI, diabetes, history of stroke/TIA, higher CHA_2_DS_2_-VASc score, nonparoxysmal AF, increased LV internal diameter end-diastole and LA diameter	Derivation cohort: ESS score: 6.1 ± 4.0 (moderate–severe SDB) vs. 5.5 ± 4.1 (no/mild SDB), *p* = 0.099 (NS)
Rogel, 2024 [78]	Not reported	Not reported
Wei, 2024 [79]	Risk factors for AF (group B and C) vs. non-AF (group A): Higher BMI, severe OSA, lower SBP, higher DBP, higher HR, larger LA diameter and volume, larger RV and atrial diameter (end-diastole and systole), larger RV outflow tract, and larger right atrial area	Not reported

Legend: AF, atrial fibrillation; AHI, apnea–hypopnea index; BMI, body mass index; CAD, coronary artery disease; CHA_2_DS_2_-VASc, Congestive heart failure, Hypertension, Age ≥ 75 (2 points), Diabetes, Stroke/TIA/thromboembolism (2 points), Vascular disease, Age 65–74, Sex category (female); CHD, coronary heart disease; CI, confidence interval; DBP, diastolic blood pressure; EDS, excessive daytime sleepiness; ep/h, events per hour; ESS, Epworth Sleepiness Scale; FOSQ, Functional Outcomes of Sleep Questionnaire h, hour; Hb, hemoglobin; HbA1c, glycated hemoglobin; HF, heart failure; HR, heart rate; ICD, International Classification of Diseases; IQR, interquartile range; LA, left atrium/atrial; LF/HF, low frequency to high frequency; LV, left ventricle/ventricular; MCA, Minimal Cross-sectional Area; MI, myocardial infarction; min, minute; MoCA, Montreal Cognitive Assessment; NC, neck circumference; NoSAS, Neck, Obesity, Snoring, Age, Sex; NS, non-significant; NSR, normal sinus rhythm; OR, odds ratio; OSA, obstructive sleep apnea; OSAHS, obstructive sleep apnea–hypopnea syndrome; OSAS, obstructive sleep apnea syndrome; PAF, paroxysmal atrial fibrillation; PAP, pulmonary artery pressure; pts, patients; RAS, Renin-Angiotensin System; RDI, respiratory disturbance index; RMSSD, root mean square of successive differences between normal heartbeats; RV, right ventricle/ventricular; SA, sleep apnea; SaO2, arterial oxygen saturation; SBP, systolic blood pressure; SD, standard deviation; SDB, sleep-disordered breathing; SDNN, standard deviation of normal to normal beat intervals; SII, systemic inflammatory index; STOP-BANG, Snoring, Tiredness, Observed apneas, high blood Pressure, BMI, Age, Neck circumference, Gender (OSA screening questionnaire); T90, time under 90% oxygen saturation; TIA, transitory ischemic accident; VAI, visceral adipose index; WP-AHI, watch-type peripheral arterial tonometry apnea–hypopnea index; yrs, years.

**Table 4:** Summary of laboratory, ECG, echocardiographic and polysomnographic findings in the included studies.

First Author, Year	Laboratory Findings	ECG and Echocardiographic Findings	Polysomnographic Findings
Caples, 2019 [46]	Not reported	No significant differences between groups (*p* > 0.05) LVEF: 57.3 ± 7.1% (control) vs. 58.1 ± 7.5% (PAP) LAVI: 35.6 ± 6.5 vs. 40.6 ± 6.8 mL/m^2^	Control vs. PAP: AHI: 29.8 ± 21 (control) vs. 30.3 ± 19.5 (PAP) events/h Obstructive Apnea Index: 13.8 ± 16.0 vs. 13.7 ± 16.1 Central Apnea Index: 0.6 ± 2.3 vs. 0.8 ± 2.3 Min SpO_2_: 80.1 ± 8.2% vs. 80.2 ± 8.1% Time with SpO_2_ > 90%: 90.2 ± 14.1% vs. 90.1 ± 14.2% (*p* > 0.9)
Feng, 2019 [23]	Not reported	Not reported	Not reported
Gonçalves, 2019 [7]	Not reported	Not reported	RDI-PM correlated with PSG (R = 0.34, *p* = 0.004); cut-off 13.3 had 78% sensitivity/specificity for OSAS, 90% sensitivity for moderate-to-severe OSAS; AF reduced specificity to 57%
Hojo, 2019 [47]	Non-OSA vs. treated OSA vs. untreated OSA: BNP: 80.4 ± 157.6 vs. 40.9 ± 24.3 vs. 154.9 ± 210.6 Cre: 0.89 ± 0.22 vs. 0.80 ± 0.10 vs. 1.3 ± 1.8 BNP (pg/mL) and Cre (g/dL) levels did not differ between groups.	No statistically significant differences in other parameters such as LA diameter and volume, EF, and E/e’, were found between groups Non-OSA vs. treated OSA vs. untreated OSA (NS): LA diameter: 37.4 ± 9.7, 41.5 ± 6.2, 41.7 ± 7.4 mm; EF: 62.7 ± 7.1%, 63.6 ± 10.3%, 62.4 ± 14.1%; E/e′: 12.8 ± 10.5, 11.4 ± 3.2, 17.6 ± 14.2 LAV: 103.7 ± 25.1 mL, 107.5 ± 30.0 mL, 120.3 ± 40.1 mL	AHI (type 3 study), events/h: 5.8 ± 3.8 non-OSA; 26.0 ± 15.8 treated OSA; 28.1 ± 10.9 untreated OSA, *p* = 0.0001 CPAP initiated after 2nd session in severe OSA cases
Linz, 2019 [48]	Not reported	Not reported	Mean RDI: 17.9 ± 11.5 events/h RDI ≥ 20/h in ≥1 night: 85% of pts; Severe SDB (mean RDI ≥ 20/h): 32% of pts; High night-to-night RDI variability (SD ≈ 6.3 events/h); RDI quartile 4 vs. quartile 1 associated with 1.7×, 2.3×, and 10.2× increased risk of AF >5 min, >1 h, and >12 h/day, respectively (*p* < 0.001 for all)
Providência, 2019 [49]	eGFR: 75.1 ± 18.4 mL/min (declined with BMI); no other laboratory values reported	Indexed LA volume: 48.6 ± 18.6 mL/m^2^ LVEF: 62 ± 9% LVEF < 35% in 2% overall	Not reported
Bazan, 2020 [50]	CHA_2_DS_2_-VASc: 1.9 ± 1.7 (higher in OSA: 2.1 ± 1.7 vs. 0.8 ± 1.4, *p* = 0.012); no other labs reported	OSA vs. non-OSA: LA diameter: 44.8 ± 5 mm vs. 43.5 ± 7 mm, *p* = NS LVEF: 57.7 ± 12% vs. 56.7 ± 10%, *p* = NS PR: 189 ± 35 ms vs. 167 ± 30 ms, *p* = 0.056 p-wave duration: 133 ± 25 ms vs. 121 ± 20 ms, *p* = 0.11	AHI ≥ 5 in 82% of pts: 26% 5 ≤ AHI < 15, 26% 15 ≤ AHI < 30, 30% AHI ≥ 30 AHI: 28 ± 22 (men) vs. 17 ± 15 (women), *p* = 0.048 STOP-BANG ≥ 4 predicted CPAP indication (OR 4.5 [1.9–10.6]) AHI correlated with BMI (r = 0.36, *p* = 0.003) and STOP-BANG (r = 0.45, *p* < 0.001)
Ben Halima, 2020 [36]	Not reported	Not reported	Mean AHI (ambulatory study): 21.6 ± 13.6 e/h
Gellert, 2020 [51]	Total cholesterol, glucose levels collected but no groupwise data reported	48 h aECG: AF prevalence 9.3%; SA associated with AF (OR 7.3, 95% CI: 3.7–14.5); increased PACs (OR 1.2–1.5) and PVCs in SA (non-significant)	SA defined by ICD-9 codes and/or self-reported diagnosis; 217/1154 had SA; no PSG or AHI data
Martí-Almor, 2020 [52]	Not reported	Not reported	SA severity (RDI ≥ 20 via pacemaker) associated with increased AF burden over 12–18 months; persistent AF: 16.9% vs. 7.3%; 15.3% treated pts; fallback mode switching > 7 d: Δ 13.4%, 95% CI: 4.1–22.7%
May, 2020 [15]	Not reported	PAF vs. controls: LAV: 64.2 [IQR 48.9, 77.4] vs. 56.2 [IQR 45.1, 70.0], *p* = 0.006 HR: 64.99 ± 10.2 bpm vs. 68.76 ± 10.4, *p* = 0.002	AHI (median [IQR]): 10.6 [3.6–23.4] (AF) vs. 12.7 [3.9–24.5] (controls), *p* = 0.33 STOP-BANG AUC = 0.75, new NABS model (neck, age, BMI, snoring) outperformed STOP-BANG (AUC = 0.88 for AHI ≥ 15)
Mazza, 2020 [53]	Not reported	EF: 58 ± 7%; LA diameter: 42 ± 5 mm	AF ≥ 6 h vs. non-AF or AF < 6 h RDI max: 58 ± 25 episodes/h vs. 49 ± 29 episodes/h, *p* = 0.001 RDI mean: 32 ± 18 vs. 30 ± 19, *p* = 0.156 RDI burden: 14% [2–33%] vs. 14% [0–36%], *p* = 0.774
Shapira-Daniels, 2020 [54]	Not reported	Not reported	Not reported (sleep study via WatchPAT)
Starkey, 2020 [55]	Not reported	Minimal cross-sectional area: 2.4 ± 0.7 cm^2^ (non-OSA) vs. 2.4 ± 0.9 cm^2^ (mild OSA) vs. 2.5 ± 1.1 cm^2^ (moderate OSA) vs. 2.2 ± 0.6 cm^2^ (severe OSA), no *p*-values reported	Not reported
Szymańska, 2020 [56]	Mean visfatin: 1.9 ± 2.1 ng/mL Visfatin (ng/mL) levels correlated with OSA severity: 1.77 ± 0.17 (mild OSA) vs. 2.38 ± 0.18 (moderate OSA) vs. 3.55± 0.61 (severe OSA), *p* for trend = 0.017	Not reported	Portable RP study (Embletta MPR) Mean AHI (all patients): 8.1 ± 10.7 events/hour
Traaen, 2020 [57]	Not reported	Not reported	ODI, SPO_2_, SaO_2_ < 90% assessed and presented by sex Values per SA severity (non-SA vs. mild SA vs. moderate SA vs. severe SA): Central Apnea Index: 0.1 (IQR 0–0.3) vs. 0.2 (IQR 0.1–0.6) vs. 0.4 (IQR 0.1–0.9) vs. 0.8 (IQR 0.2–2.8), *p* < 0.001 Central AHI > Obstructive AHI: 14% vs. 3.5% vs. 1.2% vs. 2.9%, *p* < 0.001 AHI associated with higher STOP-BANG and neck circumference
Blanchard, 2021 [58]	Not reported	Pts with incident AF vs. without incident AF: RMSSD (ms): 82 (55–121) vs. 60 (45–85), *p* < 0.0001 SDNN (ms): 71 (51–103) vs. 63 (48–84), *p* = 0.0183 pNN50 (%): 18 (11–32) vs. 18 (9–33), *p* = 0.4691 LF/HF ratio: 1 (1–1) vs. 1 (1–2), *p* < 0.0001	Pts with incident AF vs. without incident AF: AHI: 32 (18–49) vs. 22 (39–39) events/h, *p* < 0.0001 ODI 3%: 23 (11–43) vs. 14 (5–30) events/h, *p* < 0.0001 T90 (SaO_2_ < 90%): 4% (1–22) vs. 1% (0–6), *p* < 0.0001 Nadir SaO_2_: 81% (73–85) vs. 84% (78–88), *p* < 0.0001 Hypoxic burden: 52 (18–108) vs. 25 (8–62) % min/h, *p* < 0.0001
Delesie, 2021 [59]	Not reported	Not reported	Not reported
Mohammadieh, 2021 [60]	Not reported	OSA (AHI ≥ 15) vs. non-OSA (AHI < 15): LVEF: 54.6 ± 9.8 vs. 58.8 ± 7.7, *p* = 0.075 LA area: 26.6 ± 4.7 vs. 23.4 ± 5.2 cm^2^, *p* = 0.048 LA diameter: 4.3 ± 0.6 vs. 4.0 ± 0.6 cm, *p* = 0.211	OSA (AHI ≥ 15) vs. non-OSA (AHI < 15): AHI: 31.9 ± 15.5 vs. 5.2 ± 4.3, *p* < 0.001 ODI: 18.4 ± 13.1 vs. 2.1 ± 2.3, *p* < 0.001 CAI: 1.4 ± 2.5 vs. 0.2 ± 0.5, *p* < 0.001
Højager, 2022 [61]	AHI < 15 vs. AHI 15–30 vs. AHI > 30: HbA1c (mmol/mol): 36.8 ± 11.4 vs. 38.7 ± 7.5 vs. 42.3 ± 10.6, *p* < 0.001 Triglycerides (mmol/L): 1.8 ± 1.4 vs. 2.1 ± 1.2 vs. 2.4 ± 1.7, *p* = 0.019 Total cholesterol (mmol/L): 5.9 ± 5.9 vs. 5.2 ± 1.1 vs. 5.1 ± 1.0, *p* = 0.14 (NS) LDL-C (mmol/L): 3.1 ± 1.0 vs. 2.9 ± 0.9 vs. 2.8 ± 1.0, *p* = 0.07 (NS)	AHI < 15 vs. AHI 15–30 vs. AHI > 30: HR: 70.8 ± 11.5 vs. 68.8 ± 12.6 vs. 75.6 ± 14.3 bpm, *p* < 0.001	AHI: 34.2 (0.2–115.8) Central apneas (events/hour): 2.1 ± 3.3 (AHI < 15) vs. 6.0 ± 8.1 (AHI 15–30) vs. 16.8 ± 36.4 (AHI > 30), *p* < 0.001
Latif, 2022 [62]	Not reported	OSA vs. non-SA, no *p*-values reported: LA diameter: 5.0 (5–6) cm vs. 6.0 (5–6) cm EF: 57% (55–65) vs. 60% (55–65)	OSA vs. non-SA, no *p*-values reported: AHI: 4%: 9 (5–14) events/h vs. 1 (1–2) Minimum oxygen saturation (%): 89 (85–90) vs. 91 (91–92)
Mehawej, 2022 [63]	Hb: 14 ± 2 g/dL (high-risk OSA) vs. 13 ± 2 g/dL (low-risk OSA), *p* < 0.01	LVEF: 54 ± 11.4% (high-risk OSA) vs. 55 ± 11.3% (low-risk OSA), NS Heart rate: 72 ± 15.5 bpm vs. 71 ± 13.5 bpm, NS	Not reported (OSA assessed via STOP-BANG questionnaire only; no PSG or polygraphy performed)
Oster, 2022 [64]	Not reported	Not reported	OSA was screened using STOP-BANG only; no PSG or oximetry data reported
Verhaert, 2022 [65]	Not reported	Not reported	WatchPAT-based sleep parameters across SDB severity (none vs. severe): Awakenings: 7.0 [5,6,7,8,9,10] (none) vs. 12 [9,10,11,12,13,14,15,16,17] (severe), *p* = 0.01 pAHI: 3.1 [1.5–3.8] vs. 43 [33,37,38,39,40,41,42,43,44,45,46,59,60,70], *p* < 0.01 Central pAHI: 0 [0–0.2] vs. 3.0 [1.1–7.2], *p* < 0.01 ODI: 0.6 [0.3–1.0] vs. 22 [16,17,18,19,20,21,22,23,24,25,26], *p* < 0.01 SpO_2_ nadir: 92% vs. 91%, *p* < 0.01 Time SpO_2_ < 90%: 0 vs. 7.0 [0.9–16] min, *p* < 0.01 SpO_2_ < 90% (% sleep time): 0% vs. 1.2% [0.2–2.8], *p* < 0.01
Ahn, 2023 [66]	Not reported	Not reported	Not reported
Betz, 2023 [67]	Not reported	Not reported	Not reported
Holtstrand, 2023 [68]	Not reported	Not reported	Mean AHI: 25.4 ± 19.7 events/h AHI (events/h) per OSA severity (non-OSA, mild, moderate, severe): 3.0 (2.0–4.0) vs. 9.4 (7.0–12.0) vs. 20.6 (17.0–24.9) vs. 44.0 (35.1–58.0), *p* = 0.000 ODI (events/h): 1.0 (0.5–2.0) vs. 4.0 (2.0–6.0) vs. 11.0 (7.0–16.0) vs. 32.0 (21.5–47.8) *p* = 0.000 Mean SpO_2_ (%): 95.9 (94.9–96.6) vs. 95.0 (94.0–96.0) vs. 94.0 (93.0–95.0) vs. 93.0 (91.8–94.0), *p* < 0.001 Nadir SpO_2_ (%): 91.0 (88.0–92.0) vs. 88.0 (85.0–90.0) vs. 85.0 (81.0–87.0) vs. 79.0 (73.0–83.0), *p* < 0.001
Jensen, 2023 [69]	Not reported	Moderate/severe SA vs. none/mild SA: LVEF: 55.0 (50.0–60.0) vs. 55.0 (55.0–60.0) *p* = 0.016 LA dilatation: 46.5% vs. 32.7%, NS	Moderate-to-severe SA vs. none/mild SA: AHI: 30.0 (20.0–38.0) vs. 7.0 (5.0–11.4), *p* < 0.001 ODI < 3% (events/hour): 27.0 (17.0–35.0) vs. 8.0 (6.0–11.0), *p* < 0.001 ODI < 4% (events/hour): 18.0 (10.6–26.5) vs. 4.0 (3.0–6.0), *p* < 0.001 SpO_2_ minimum (%): 79 (74–84) vs. 85 (78–87), *p* < 0.001
Lin, 2023 [70]	Total cholesterol: 3.7 ± 1.1 mmol/L (OSAHS) vs. 4.9 ± 1.2 mmol/L (non-OSAHS), *p* = 0.003 LDL-C: 2.5 (2.0–2.9) mmol/L (OSAHS) vs. 3.2 (2.4–4.2) mmol/L (non-OSAHS), *p* = 0.022 Triglycerides, creatinine, uric acid, and glucose also assessed, not significant	Not reported	AHI: 20.3 (12.5–31.9) events/h (OSAHS) vs. 2.9 (1.4–3.5) events/h (non-OSAHS), *p* < 0.001
Mills, 2023 [71]	Not reported	Not reported	Non-OSA, mild, and moderate-to-severe OSA, no *p*-values reported: pAHI 3%: 3.15 (2.8–4.0) vs. 10.75 (7.9–11.7) vs. 32.6 (19.6–45.2) pAHI 4%: 0.85 (0.8–1.1) vs. 2.75 (2.2–4.8) vs. 14.8 (10.1–26.8) ODI 3%: 3.05 (2.5–3.4) vs. 10.4 (7.35–11.8) vs. 31.4 (18.6–45.0) Time SpO_2_ < 90% (min): 0.03 (0.0–0.10) vs. 3.40 (0.0–44.9) vs. 8.16 (0.0–40.8) Central Sleep Apnea Index: 0.1 (0.0–0.23) vs. 0.65 (0.0–1.0) vs. 1.75 (1.15–3.5)
Ozkan, 2023 [72]	AF vs. non-AF (NSR): HDL-C: 39 (30–45) vs. 41 (28–53), *p* = 0.003 Triglycerides: 213 (174–267) vs. 197 (144–255), *p* = 0.001 Lower LDL-C: 143.5 ± 17.8 vs. 152.3 ± 14.9, *p* < 0.001 Cre levels: 1.1 ± 0.2 vs. 1 ± 0.2, *p* = 0.02 Platelet count: 232.7 ± 32.3 vs. 211.2 ± 31.4, *p* < 0.001 Lymphocyte count: 1.8 (1.3–2.3) vs. 2.2 (1.3–3.1), *p* < 0.001 Neutrophil count: 5 ± 1 vs. 5.4 ± 1.5, *p* = 0.03 VAI: 8 (5.3–15.5) vs. 6.7 (3.8–14.6), *p* < 0.001 SII: 624.4 (284.5–950.6) vs. 539.4 (238.7–1131.0), *p* < 0.001	AF vs. non-AF (NSR): EF: 58 (51–69) vs. 57 (50+), *p* = 0.013 PAP: 38 (27–46) vs. 35 (4–46), *p* < 0.001	AF vs. non-AF (NSR): AHI: median 27 (21–33) vs. 23 (8–36), *p* < 0.001
Rivas, 2023 [73]	Not reported	Not reported	Not reported
Tanaka, 2023 [74]	Not reported	LVDd: 47.7 ± 5.0 mm; LAD: 40.8 ± 6.1 mm; LVEF: 63.5 ± 10.5%; LAD ≥ 40 mm: not an independent predictor of CPAP indication (multivariate OR 1.37, *p* = 0.23)	WP-AHI: 25.9 ± 12.7; PSG-AHI: 31.4 ± 18.9; correlation r = 0.48, *p* < 0.001; 35.7% of patients with WP-AHI < 30 had PSG-AHI ≥ 30; WP-AHI ≥ 18.1 best cutoff to predict PSG-AHI ≥ 20 (AUC 0.72)
Vermeer, 2023 [75]	Total and LDL-cholesterol, HbA1c, fasting glucose assessed at baseline and follow-up; no results yet reported (protocol paper)	ECG and echocardiographic parameters part of standard care, but not specified; to be assessed pre- and post-ablation	OSA screening by WatchPAT™ 300; OSA suspected if AHI ≥ 5; no AHI, ODI or SpO_2_ values reported
Hunt, 2024 [76]	Baseline: Mean Hb: 14.7 ± 1.1 g/L Mean Cre: 79 ± 15 µmol/L NT-proBNP: 140 ng/L (IQR 205)	Not reported	AHI: 27 (IQR 15–86) ODI: 27 (IQR 13–90)
Kadhim, 2024 [77]	Not reported	Moderate-to-severe SDB (AHI ≥ 15) vs. no/mild SDB (AHI < 15) (derivation cohort): LVEF (%): 60.5 ± 10.4 vs. 61.0 ± 8.0, *p* = 0.056 LVIDd (cm): 5.1 ± 0.8 vs. 4.9 ± 0.6, *p* = 0.001 LA diameter (cm): 4.2 ± 0.6 vs. 3.9 ± 0.6, *p* < 0.001 LA volume index (mL/m^2^): 34.3 ± 11.3 vs. 32.0 ± 11.0, *p* = 0.053 (borderline)	Validation cohort: AHI: 35.9 ± 20.1 (moderate–severe SDB) vs. 7.2 ± 3.9 (no/mild SDB), *p* < 0.001
Rogel, 2024 [78]	Not reported	Not reported	Not reported
Wei, 2024 [79]	BNP (pg/mL): Group A, 674 ± 132 Group B, 752 ± 145 (*p* < 0.05 vs. A) Group C, 1053 ± 156 (*p* < 0.05 vs. A & B) (*p* < 0.001).	AF (group B and C) vs. non-AF (group A): Left heart: LAD: 52.5 ± 6.0 vs. 38.7 ± 5.7 mm, *p* < 0.001; LAV: 105 ± 39 vs. 87 ± 34 mL, *p* = 0.001; LVEF: 63 ± 15 vs. 69 ± 16%, *p* = 0.006; FS: 31 ± 15 vs. 38 ± 14%, *p* = 0.001; E/A: 1.29 ± 0.48 vs. 0.98 ± 0.29, *p* < 0.001; e′: 5.3 ± 1.8 vs. 6.6 ± 1.8 cm/s, *p* < 0.001; E/e′: 10.9 ± 3.9 vs. 14.3 ± 3.5, *p* < 0.001; PV-D: 58 ± 20 vs. 45 ± 20 cm/s, *p* < 0.001; PV-S: 45 ± 20 vs. 53 ± 20 cm/s, *p* < 0.001; FPV: 53 ± 27 vs. 42 ± 24 cm/s, *p* = 0.003. Right heart: RV D1: 38.1 ± 6.3 vs. 34.2 ± 5.7 mm, *p* < 0.001; RV D2: 30.7 ± 5.4 vs. 27.6 ± 4.4 mm, *p* < 0.001; RVOT2: 30.3 ± 3.4 vs. 23.2 ± 3.2 mm, *p* < 0.001; RA D1: 46.9 ± 7.1 vs. 39.5 ± 5.9 mm, *p* < 0.001; RA D2: 40.4 ± 6.1 vs. 34.6 ± 5.2 mm, *p* < 0.001; RA area: 26.7 ± 7.1 vs. 17.3 ± 5.9 cm^2^, *p* < 0.001.	Not reported

Legend: AF, atrial fibrillation; AHI, apnea–hypopnea index; aECG, ambulatory electrocardiogram; AUC, area under the curve; BMI, body mass index; BNP, B-type natriuretic peptide; CAD, coronary artery disease; CAI, Central apnea index; CI, confidence interval; CPAP, continuous positive airway pressure; Cre, creatinine; DBP, diastolic blood pressure; EDS, excessive daytime sleepiness; EF, ejection fraction; e′, early diastolic mitral annular velocity; e/h, events per hour; ESS, Ep-worth Sleepiness Scale; FPV, flow propagation velocity; FS, fractional shortening; h, hour; Hb, hemoglobin; HbA1c, glycated hemoglobin; HDL-C, high-density lipoprotein cholesterol; HF, heart failure; HR, heart rate; ICD, International Classification of Diseases; IQR, interquartile range; LAD, left atrial diameter; LAV, left atrial volume; LAVI, left atrial volume index; LDL-C, low-density lipoprotein cholesterol; LF/HF, low-frequency/high-frequency (heart rate variability) ratio; LVEF, left ventricular ejection fraction; LVIDd, left ventricular internal diameter in diastole; min, minutes; MoCA, Montreal Cognitive Assessment; ms, milliseconds; NoSAS—Neck, Obesity, Snoring, Age, Sex (screening tool); NS, not significant; NSR, normal sinus rhythm; NT-proBNP, N-terminal prohormone of brain natriuretic peptide; OAI, obstructive apnea index; ODI, oxygen desaturation index; OR, odds ratio; OSA, obstructive sleep apnea; OSAHS, obstructive sleep apnea–hypopnea syndrome; OSAS, obstructive sleep apnea syndrome; PAF, paroxysmal atrial fibrillation; PAP, pulmonary artery pressure; PACs, premature atrial contractions; pAHI, peripheral apnea–hypopnea index; PVCs, premature ventricular contractions; PR, PR interval; PSG, polysomnography; pts, patients; pNN50, percentage of successive RR intervals differing by >50 ms; PV-D, pulmonary vein diastolic flow velocity; PV-S, pulmonary vein systolic flow velocity; pRDI, respiratory disturbance index (with ≥3% desaturation); RA area, right atrial area; RA D1, right atrial major diameter; RA D2, right atrial minor diameter; RDI, respiratory disturbance index; RDI-PM, RDI measured by peripheral monitoring; RMSSD, root mean square of successive differences (HRV); RV D1, right ventricular basal diameter; RV D2, right ventricular mid-cavity diameter; RVOT2, right ventricular outflow tract (subcostal view); RV, right ventricle; SA, sleep apnea; SAM, sleep apnea monitoring; SaO_2_, arterial oxygen saturation; SBP, systolic blood pressure; SD, standard deviation; SDNN, standard deviation of NN intervals; SDB, sleep-disordered breathing; SII, systemic immune–inflammation index; SpO_2_, peripheral oxygen saturation; STOP-BANG, screening tool: Snoring, Tiredness, Observed apnea, high BP, BMI, Age, Neck, Gender; T90, time with SpO_2_ < 90%; TIA, transient ischemic attack; VAI, visceral adiposity index; WP-AHI, WatchPAT-derived apnea–hypopnea index; yrs, years.

## Supplementary Materials

The following supporting information can be downloaded at: https://www.mdpi.com/article/10.3390/jcm14165708/s1, Table S1: PRISMA 2020 for Checklist (1); Table S2: PRISMA 2020 for Abstracts Checklist (2) [113].
